# A Genome-Wide Analysis of the *Pentatricopeptide Repeat* (PPR) Gene Family and PPR-Derived Markers for Flesh Color in Watermelon (*Citrullus lanatus*)

**DOI:** 10.3390/genes11101125

**Published:** 2020-09-24

**Authors:** Saminathan Subburaj, Luhua Tu, Kayoun Lee, Gwang-Soo Park, Hyunbae Lee, Jong-Pil Chun, Yong-Pyo Lim, Min-Woo Park, Cecilia McGregor, Geung-Joo Lee

**Affiliations:** 1 Department of Horticulture, Chungnam National University, Daejeon 34134, Korea; sami_wheat@cnu.ac.kr (S.S.); luluguniang@gmail.com (L.T.); kayoun200@cnu.ac.kr (K.L.); kps21641001@hanmail.net (G.-S.P.); lhb7982@gmail.com (H.L.); jpchun@cnu.ac.kr (J.-P.C.); yplim@cnu.ac.kr (Y.-P.L.); 2 Department of Smart Agriculture Systems, Chungnam National University, Daejeon 34134, Korea; 3 Breeding Institute, Hyundai Seed Co Ltd., Yeoju, Gyeonggi-do 12660, Korea; p.minwoo@gmail.com; 4 Department of Horticulture, University of Georgia, Athens, GA 30602, USA; cmcgre1@uga.edu

**Keywords:** watermelon, pentatricopeptide-repeat (PPR) gene family, comprehensive analysis, expression profiling, flesh color

## Abstract

Watermelon (*Citrullus lanatus*) is an economically important fruit crop grown for consumption of its large edible fruit flesh. *Pentatricopeptide-repeat* (PPR) encoding genes, one of the large gene families in plants, are important RNA-binding proteins involved in the regulation of plant growth and development by influencing the expression of organellar mRNA transcripts. However, systematic information regarding the PPR gene family in watermelon remains largely unknown. In this comprehensive study, we identified and characterized a total of 422 *C. lanatus* PPR (*ClaPPR*) genes in the watermelon genome. Most *ClaPPRs* were intronless and were mapped across 12 chromosomes. Phylogenetic analysis showed that *ClaPPR* proteins could be divided into P and PLS subfamilies. Gene duplication analysis suggested that 11 pairs of segmentally duplicated genes existed. In-silico expression pattern analysis demonstrated that *ClaPPRs* may participate in the regulation of fruit development and ripening processes. Genotyping of 70 lines using 4 single nucleotide polymorphisms (SNPs) from 4 *ClaPPRs* resulted in match rates of over 0.87 for each validated SNPs in correlation with the unique phenotypes of flesh color, and could be used in differentiating red, yellow, or orange watermelons in breeding programs. Our results provide significant insights for a comprehensive understanding of *PPR* genes and recommend further studies on their roles in watermelon fruit growth and ripening, which could be utilized for cultivar development of watermelon.

## 1. Introduction

Pentatricopeptide (PPR) proteins are one of the largest gene families in plants, and are usually characterized by an array of 2–30 tandem repeats of a degenerated unit consisting of 30–40 amino acid (aa) sequence motifs [1]. According to the domain architecture, PPR proteins are divided into subfamilies of P and PPR-like long and short (PLS), which are characterized by motifs without space and motifs with interspaced either of short (31 aa) or long (35–36 aa) PPR-like motifs, respectively. Based on the domain assembly in the C terminal of a PPR protein, the PLS subfamily is further classified into five subgroups: PLS, E1, E2, E+, and DYW [1,2,3]. Since the discovery of PPR proteins in yeast (*Saccharomyces cerevisiae* L.) [4], these have been reported in various terrestrial plants. Being a large gene family, more than 400 PPR proteins have been reported in various plants, including *Arabidopsis* (441), foxtail millet (486), poplar (626), maize (491), and rice (477) [1,3,5,6,7]. PPR proteins have been found to exhibit RNA-binding properties, which facilitate in mediating gene expression through posttranscriptional processes associated with transcripts in the mitochondria, chloroplast, and nucleus. Thus, PPR genes are thought to have a major impact on organelle stability, including biogenesis, and function through their involvement in various posttranscriptional processes such as RNA-editing [1,8], RNA-splicing [9], and RNA-processing [10].

The functions of PPR proteins have been reported to be associated with plant growth and development and organelle formation. In Arabidopsis, a chloroplast-localized PPR protein, called EMBRYO-DEFECTIVE175 (EMB175), has been found to influence embryo morphogenesis [11]. Similarly, in maize, a mitochondria-localized P-type PPR protein, EMPTY PERICARP12 (EMP12), has been reported to be essential for embryogenesis and endosperm development through trans-splicing of mitochondrial *nad2* introns [12]. Hsieh et al. [13] showed that the *SLOW GROWTH3* (*SLO3*) gene, encoding a PPR protein, was involved in the splicing of *nad7* intron 2 in Arabidopsis, and its mutant, *slo3,* exhibited a dysfunctional mitochondrion, which resulted in growth retardation and delayed development. In rice, few DYW-type PPR proteins, such as OPAQUE AND GROWTH RETARDATION 1 (OGR1) and PPS1, play important roles in C→U RNA editing of mitochondrial transcripts; upon silencing of these genes using T-DNA insertion and RNAi, mutant plants exhibited various pleiotropic phenotypes, including late seed germination, retarded growth, delayed development, dwarfing, and partial pollen sterility at both vegetative and reproductive stages [14,15]. In addition to plant developmental process, PPR proteins have been reported to be involved in the responses to biotic and abiotic stresses. For instance, in Arabidopsis, several PPR proteins such as *SOAR1* [16], *PGN* [17], *SLG1* [18], and *PPR96* [19], have been shown to participate in responses to abiotic stresses. 

PPR proteins have also been characterized to be involved in cytoplasmic male sterility (CMS); it is an important intriguing issue in plants [20], as only the male gametes are impaired, resulting in a failure to produce functional pollens. Some of the PPR proteins encoded as restorers of fertility (*Rf*) genes mask the mitochondrial transcripts that cause CMS and thus restores fertility. The *Rf*-*PPR* genes have been reported in various terrestrial plants, including petunia (*RF952*) [21], radish (orf687) [22], pepper (*CaPPR6*) [23], and Arabidopsis (*RFL2*) [24]. Increasing molecular evidence has clearly emphasized the roles of PPR genes in fruit development, ripening, and flesh color of plants [25,26,27]. *GUN1,* which encodes a plastid-located PPR protein, has been reported to be involved in the plastid-to-nucleus retrograde signaling pathway during fruit development and ripening in tomato [26]. In relation to this, ripening impaired tomato mutants such as *Cnr* dramatically repressed expression of genes associated with ripening, cell wall-degrading enzymes and PPR repeat-containing proteins, resulting in mature fruits with colorless pericarp tissue, thereby indicating that PPR proteins play a significant role in fruit development [25]. Recently, in melon (*Cucumis melo* L.), the *white-flesh* gene, named *CmPPR1* (MELO3C003069), encoding a plastid-targeted P-type PPR protein, has been reported to be a candidate gene in one of the two major QTL, which determine flesh color intensity [27]. Furthermore, the polymorphic SNP markers in P-class motifs of *CmPPR1* have been found to contribute to genetic variation in orange, green, and white fruit flesh colors within the species. It has also been established that this *CmPPR1* is possibly involved in plastid-to-nucleus retrograde signaling, thereby affecting the expression of plastid-targeted genes, indicating the involvement of PPR proteins in fruit flesh color variation [27].

Watermelon (*Citrullus lanatus*) is an important fruit crop with overall annual production of more than 103 million tons worldwide (http://www.fao.org/statistics/en/. Watermelon exhibits diverse variation in fruit-quality traits, including soluble sugars, firmness, fruit size, shape, skin, and flesh color, along with functional factors such as lycopene and β-carotene [28,29]. This extensive polymorphic variation motivates researchers to investigate the genetics of watermelon fruit-quality traits. PPR genes related to fruit development and flesh color variation in watermelon have not been studied yet. Fortunately, the recently released watermelon (97103 v2) genome sequence (http://cucurbitgenomics.org) provides an excellent opportunity to perform a genome-wide analysis of important gene families, including the PPR gene family in watermelon. In the present study, we identified and characterized 464 putative *PPR* genes from 97103 watermelon genome. Furthermore, we investigated their intron-exon organization, chromosomal localization, types of PPR motifs, functional diversification, subcellular locations, and phylogenetic analysis. With a focus on the involvement of PPR genes in fruit development and flesh color variation in watermelon, we also examined their expression patterns through several RNA-seq analyses from the cucurbit expression atlas (http://cucurbitgenomics.org/rnaseq/home). Finally, diagnostic SNP-CAPS markers of PPR genes were developed to study their association with fruit flesh color variation. Thus, the findings of this study will contribute to the understanding of PPR gene distribution and functions in watermelon, and also improve our understanding of the relationship between PPR genes and flesh color variations.

## 2. Materials and Methods

### 2.1. Plant Materials

All the watermelon lines used in this study were obtained from domestic seed companies (Hyundai Seed Company, Gyeonggi-do, South Korea) in Republic of Korea. A total of 70 lines with red (33), yellow (17), and orange (20) flesh colors were used in this study (Table S1). Seeds of the lines were sown in 72-cell polyethylene flats and cultivated under greenhouse at 25 and 20 ºC under 16 and 8 h light and dark conditions, respectively, until the appearance of second and third true leaves. Thereafter, the leaf samples were collected and the genomic DNA from the leaves were isolated using the WizPrep™ Plant DNA Mini Kit (Wizbiosolutions, Seongnam, South Korea).

### 2.2. Sequence Retrial and Identification of the PPR Family Members in Watermelon

To retrieve PPR genes in watermelon, the PPR motif ‘‘PF01535” from the Pfam (http://pfam.sanger.ac.uk/) database was used to BlastP searches against Watermelon *Citrullus lanatus* subsp. *vulgaris* cv. 97103 protein (version 2) sequences on the cucurbit genomics database (CuGenDB; http://cucurbitgenomics.org). Additionally, a BlastP search was also investigated with Arabidopsis and rice PPR proteins [1,7]. As queries (e-value set at1 × 10^-5^) against watermelon (version 2) protein sequences. Apart from Blastp analysis, ‘*Pentatrichopeptide repeat*’ was used as a keyword in a functional annotation search in the genome of Watermelon (97103) version 2 at the CuGenDB. After combining the results from above searches, redundant sequences were removed. The non-redundant PPR proteins in watermelon with the presence of PPR motif with confidence (E-value < 0.1) in SMART (http://smart.embl-heidelberg.de/) were taken into further analysis. To analyze the protein structure and PPR motif types in translated PPR protein sequences, the HMMsearch program from the HMMER package [30] was used to classify P or PLS (E1, E2, E+, and DYW). The PPR proteins with zero or less than 2 P motifs were excluded from further analysis following reports of previous studies on rice and cotton [7,31]. Finally, the identified candidate PPR genes were named as *Citrullus lanatus* PPR (*ClaPPR*).

### 2.3. Chromosomal Locations, Genomic Distribution, Exons/intron Organization, and Synteny Analysis

Information on accession number, chromosomal locations, CDS, and protein sequences of each non-redundant PPR gene of watermelon 97103 (version 2) were finally retrieved from the cucurbit genomics database (CuGenDB; http://cucurbitgenomics.org). The identified *ClaPPR* genes were mapped proportionally on watermelon chromosomes using CIRCOS image software [32]. The exon/intron organization of *ClaPPR* genes were predicted and investigated using the Gene Structure Display Server (GSDS2.0; http://gsds.cbi.pku.edu.cn/). To analyze the gene duplication and syntenic relationship of *ClaPPR* genes, the multiple Collinearity Scan toolkit (MCScanX) was employed with default parameters as previously reported [33]. Watermelon 97103 (version 2) genes were classified into segmental or tandem duplication types using an all-against-all BLASTP comparison (e-value ≤1 × 10^−10^). Putative segmentally duplicated gene pairs of *ClaPPR* in watermelon genome were visualized in CIRCOS. To evaluate the selection pressure on duplicated *ClaPPR* genes, the rates of non-synonymous (Ka) and synonymous substitution (Ks) were determined using the PAL2NAL web program (http://www.bork.embl.de/pal2nal/).

### 2.4. Gene Ontology (GO), Motif Identification, and Subcellular Location Prediction

The *ClaPPR* genes were Blast searched against the Arabidopsis genome (https://www.arabidopsis.org/Blast/cereon.jsp), and corresponding Arabidopsis homolog accessions (selected at an E-value of1 × 10^-10^) for each *ClaPPR* were retrieved. GO annotations were performed for the *ClaPPR* using the Arabidopsis accessions. AgriGO web-based tool (v1.2)153 was used for gene ontology (GO) enrichment analysis (*p*  <  0.05) of *ClaPPR* genes (http://systemsbiology.cau.edu.cn/agriGOv2/index.php) [34]. The conserved motifs among *ClaPPR* proteins were investigated with the following parameters: motif width between 13–50 residues, maximum number of 25 motifs, and remain parameters at default, using the MEME (Multiple Em for Motif Elicitation) software version 5.5.1 (http://meme-suite.org) [35]. The subcellular distribution of *ClaPPR* proteins were predicted using TargetP 2.0 (https://services.healthtech.dtu.dk/service.php?TargetP-2.0) and Predotarv.1.04 [36] with default parameters.

### 2.5. Phylogenetic Analysis of PPR Proteins

A total of 422 PPR protein sequences from watermelon and 44 typical PPR proteins from Arabidopsis, which utilized were in a previous study [3], were used to build a phylogenetic evolutionary tree. The translated sequences of the whole coding regions of PPR proteins were aligned using the MUSCLE method. The tree was constructed using the neighbor-joining (NJ) method with MEGA X software [37] and bootstrap analysis of 1000 replicates.

### 2.6. Expression Pattern of ClaPPR Genes in Watermelon

To study the expression patterns of *ClaPPR* genes in 97103 watermelon, the transcriptome RNA-seq data (BioProject: SRP012849) from the cucurbit expression atlas (http://cucurbitgenomics.org/rnaseq/home) were used [38]. These RNA-seq data contained data on fruit flesh (FF) and fruit rind (FR) at four stages (10, 18, 26, and 34 days after pollination (DAP)) during the development of the watermelon cultivar 97103. Furthermore, to examine the contribution and regulatory roles of *ClaPPR* genes, which are involved in fruit ripening, among different flesh-colored watermelons, the comparative watermelon transcript data (BioProject: PRJNA338036), containing five different fruit ripening stages (10, 18, 26, 34, and 42 DAP) of a pale yellow-flesh cultivated watermelon (‘COS’) and red-flesh cultivated watermelon (‘LSW-177’), were used. DEG (differentially expressed gene) values for corresponding *ClaPPR* were obtained using the list of *ClaPPR* accession numbers. The obtained DEG values were log2 base transformed. The visualization of expression in heatmap and hierarchical clustering was investigated using Cluster 3.0 and TreeView software [39].

### 2.7. Identification of Single Nucleotide Polymorphisms (SNPs) for ClaPPR Genes and Match Rate Analysis with Flesh Color

Single-nucleotide polymorphisms (SNPs) for *ClaPPR* genes in different flesh-colored (red, yellow, and orange) watermelons were identified from the whole genome resequencing (WGRS) data (Bioproject: PRJNA516776) of our recent study [29]. The SNP variant calling procedure was carried out according to our recent study [29] in which the reads of different flesh-colored watermelons from WGRS were mapped on to the watermelon 97103 reference genome. From this SNP variant calling, we searched for the SNPs that were specific to either of the flesh types such as red, yellow, and orange in the SNP subset matrix by identifying protein-coding *ClaPPR* genes bearing SNPs that were 1) monomorphic among a chosen flesh color-type, 2) monomorphic among other unchosen flesh color-types, and 3) polymorphic between a chosen and an unchosen flesh color-types. Then, the sequences of selected *ClaPPR* SNP variants were converted into CAPS markers using SNP2CAPS software [40] and simultaneously, primers were designed using Primer3Plus software (http://www.bioinformatics.nl/primer3plus). To validate CAPS markers, genomic DNA extraction, PCR assays, restriction digestion of PCR amplicons, and match rate analyses were carried out according to a previous study [29].

## 3. Results

### 3.1. Genome-Wide Identification, Classification, and Conserved Motif Analysis of PPR Genes in Watermelon

**Figure 1:**
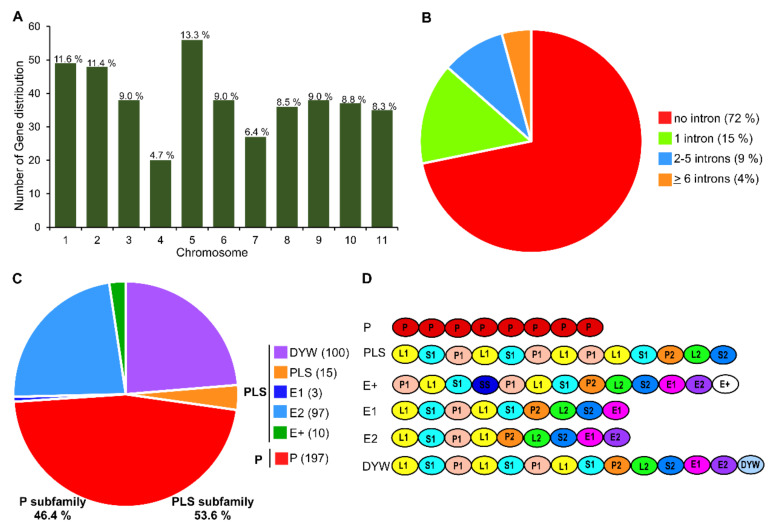
Number, distribution, and structures of *Pentatricopeptide-repeat* (*PPR*) genes in watermelon. (**A**) Number of *ClaPPR* genes in each chromosome. (**B**) Number of introns in *ClaPPR* genes. (**C**) Number of *ClaPPR* proteins belonging to the P subfamily and PPR-like long and short (PLS) subfamily with subgroups. (**D**) Typical motif structures of *ClaPPR* proteins from different subfamilies and subgroups.

Genome-wide search analysis revealed that there were 464 putative PPR genes present in watermelon genome (97103 v2). After analyzing the domain and P motif patterns, a total of 422 PPR genes were predicted and identified in this study. These watermelon PPRs were designated as *Citrullus lanatus* PPR (*ClaPPR*) from *ClaPPR1* to *ClaPPR422* proteins in the order of their chromosomal position and accession number in the CuGenDB database (http://cucurbitgenomics.org). The chromosomal location, accession number, length of ORF and protein, and number of introns are listed in Table S2. The identified number of *ClaPPR* genes on each chromosome ranged from 20 to 56. (Figure 1A). The determined exon-intron organization of 422 *ClaPPR* genes showed that 71.8% of them were intronless (303/422), while the remaining were with 1 intron (14.7%), 2–5 introns (9.2), and ≥6 introns (4.3%) as shown in Figure 1B. Analysis of repeated motifs structures in *ClaPPRs* indicated that they could be classified into P and PLS subfamilies, containing approximately equal number of genes, representing 46.4% (197 of 422) and 53.6% (225 of 422) PPR proteins, respectively (Figure 1C). In the PLS subfamily, DYW and E2 subgroups both accounted for almost half of the genes (representing 100 of 197 and 97 of 197, respectively), followed by PLS (15), E+ (10), and E1 (3) with the least number of *ClaPPR* genes (Figure 1C). Based on the tandem array of PPR motifs, in watermelon, the estimated number of PPR motifs per protein characterized was 3–27 motifs. A basic motif organization of several typical *ClaPPR* proteins representing the subfamily and subgroups are shown in Figure 1D. A strong peak was noted in the distribution at around 7–12 and 13–17 PPR motifs in P- and PLS-class proteins of watermelon, respectively (Table S2).

Analysis of conserved motifs in PPR proteins have been suggested to rule out the common molecular functions of PPR genes in different subgroups [6]. Therefore, we investigated the conserved motifs in *ClaPPR* proteins using MEME Suite (Figure S1), and results indicated that there were 25 motifs in the 422 *ClaPPR* proteins (Figure S2). Almost all of the *ClaPPR* proteins contained 16 of the 25 motifs except these nine motifs (Motifs 3, 5, 7, 13, 17, 20, 21, 24, and 25), indicating that these *ClaPPR* proteins might have a conserved domain. In addition, the majority of these motifs were analyzed mostly in P, DYW, and E2 (197, 100, and 97 *ClaPPR* genes, respectively) subgroups because they were more dominant in total number than others: E+, E1, and PLS were least in number (Figure 1C). We also found out that the different subgroups possessed specific motifs; for example, motif 21 and 25 exist only in the P subfamily. In the PLS subfamily, the DYW subgroup mainly contained motif 5, 13, and 20. Similarly, motif 24 was mainly present in the E2 subgroup. Some motifs were found to be conserved in two subgroups, such as motif 3, 7, and 17, which mainly exist in DYW and E2 subgroups (Figure S2). 

### 3.2. Chromosomal Distribution and Duplication of PPR Members in Watermelon

**Figure 2:**
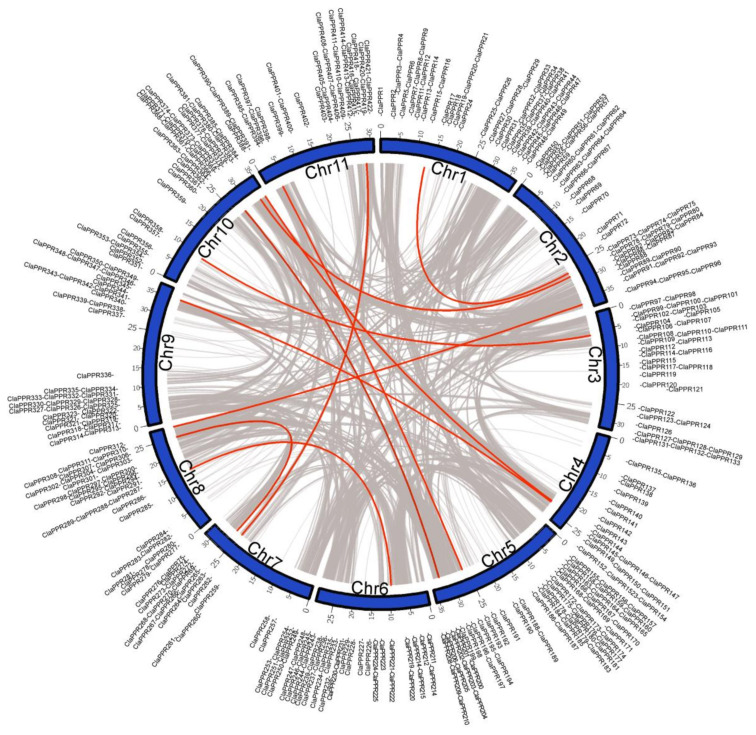
Putative chromosomal localization and gene duplication of *ClaPPRs* in watermelon. Collinear blocks in whole watermelon genome are indicated by grey lines, while the distributions of segmentally duplicated *ClaPPR* pairs are connected with red lines.

To investigate the chromosomal distribution of *ClaPPR* genes, the detailed position of *ClaPPR1*–*422* genes on watermelon (97103 v2) chromosomes were obtained from the CuGenDB database. The results showed that the identified 422 *ClaPPR* genes were distributed unevenly and widely in all the 12 chromosomes; for example, chromosome 5 and 4 were found to have the largest and fewest *ClaPPR* genes at 13.3% and 4.7%, respectively (Figure 1A and Figure 2). PPR genes usually appear in clusters or individually on chromosome [3,5]. In the present study, most of the *ClaPPR* genes clustered together either proximally or distally with very few *ClaPPR* genes positioned in the pericentromeric region of the chromosomes, indicating gene duplications during evolution. 

Duplication events of whole genome (tandem) or segmental had been portrayed as a major factor responsible for the expansion of a gene in gene families, including PPR and their subsequent evolution in plants [5,41]. Therefore, we investigated gene duplication events to determine the expansion mechanism of the *ClaPPR* members in watermelon. A total of 11 segmentally duplicated *ClaPPR* gene pairs (5 from the P subfamily and 6 from the PLS subfamily) were identified in the watermelon genome (Table S3). All the gene pairs were inter-chromosomal, involving two different chromosomes (Figure 2). Further analysis showed that the PLS subfamily consisted of three special duplicated gene pairs involving different subgroups: *ClaPPR205*, *ClaPPR264*, and *ClaPPR221*, which belong to DYW and *ClaPPR366* (E2), *ClaPPR307* (E+), and *ClaPPR286* (E2) of the E-subgroups (Table S3). In addition, the calculated ratio of non-synonymous (Ka) and synonymous (Ks) substitution ratios (Ka/Ks or ω) for these 11 duplicated gene-pairs were found to be ω < 1, suggesting that these duplicated *ClaPPR* gene pairs were under purifying selection.

### 3.3. Phylogenetic Analysis of PPR Members in Watermelon

**Figure 3:**
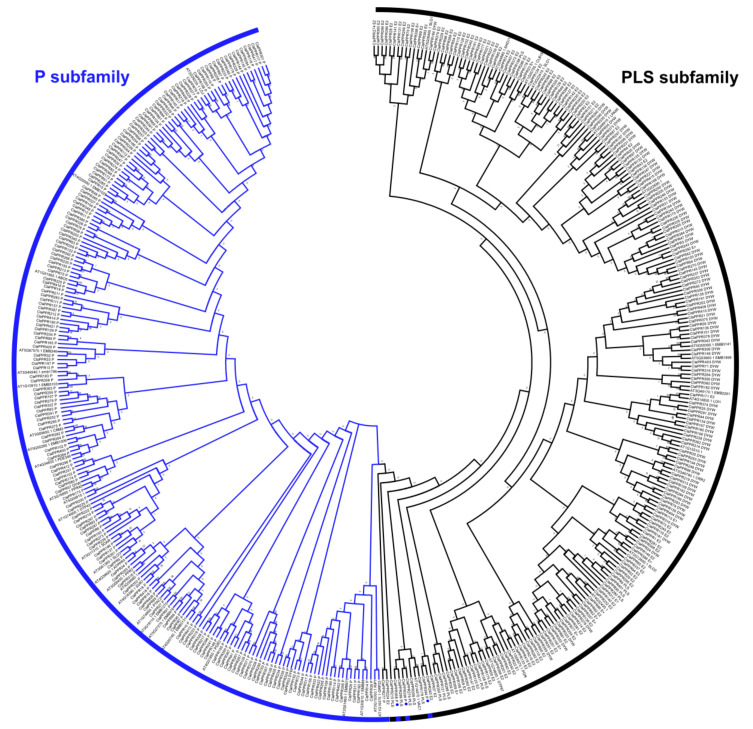
Phylogenetic relationships among the *ClaPPR* family genes. The full coding amino acid sequences of 422 *ClaPPR* proteins and 44 PPR proteins from Arabidopsis were aligned, and the NJ tree was built with 1000 bootstrap replicates using MEGA7.0. P; PLS subfamilies are represented using blue and black lines, respectively. P subfamily members which were clustered into the PLS subfamily members are indicated by a dot (blue) symbol.

In order to determine the evolutionary relationships among the *ClaPPR* family members, we constructed a phylogenetic tree based on the deduced full-length amino acid sequences of the 422 ClaPPR proteins along with the 48 PPR proteins from Arabidopsis. As expected, the tree was divided into two distinct clusters: one containing the P subfamily and the other containing the PLS subfamily (Figure 3). Interestingly, the PLS subfamily member *ClaPPR53* was clustered into the P subfamily members; similarly, several P subfamily members, including *ClaPPR338*, *ClaPPR368*, and *ClaPPR394*, were clustered into the PLS subfamily members regardless of the corresponding structure of their repeated motifs. This finding is consistent with the findings from the phylogenetic analysis of PPR proteins in rice and poplar, where P or PLS subfamily members of PPR proteins were found to be clustered into their opposite members [3,7]. These deviations in clustering could be explained by the shared structural similarities of the C-terminal motifs between P and PLS members which might have arisen via duplication of PPR motif coding regions during evolution of the aforementioned plant species including watermelon.

### 3.4. Predicted Subcellular Localization of PPR Proteins

Increasing molecular evidence suggested that PPR proteins play a pivotal role in RNA editing of transcripts in mitochondria and chloroplast organelles. Therefore, we determined the subcellular location of *ClaPPR* proteins in watermelon using TragetP2.0 and Predotar v.1.04 programs. The TragetP2.0 results showed that approximately 32% *ClaPPR* proteins were targeted to mitochondria and 6% to chloroplast, whereas Predotar v.1.04 results showed that approximately 44% and 16% were targeted to mitochondria and chloroplast, respectively (Table S4). Combining the results from both programs, we were able to predict that approximately 65% of *ClaPPR* proteins were targeted to the organelles of chloroplast (73 of 422) and mitochondria (204 of 422); however, few proteins were found to be targeted to ER (5%), and the remaining (30%) protein distributions were uncertain. In the P subfamily, almost half of the proteins (106 of 196) were predicted to be in the mitochondria and 18% (36 of 197) were in the chloroplast (Figure S3). Similarly, in the PLS subfamily, the DYW, E2, and E+ subgroup proteins had a similar localization with almost half in the mitochondria (43–50%) and some proportions in the chloroplasts (19%, 10%, and 30%, respectively). As in the case of PLS and E1, 47% and 67% were predicted in uncertain and mitochondrial localizations, respectively, and approximately 33% of both PLS and E1 proteins were targeted in the chloroplast and ER, respectively (Figure S3).

### 3.5. Gene Ontology (GO) Annotation of ClaPPR Genes

**Figure 4:**
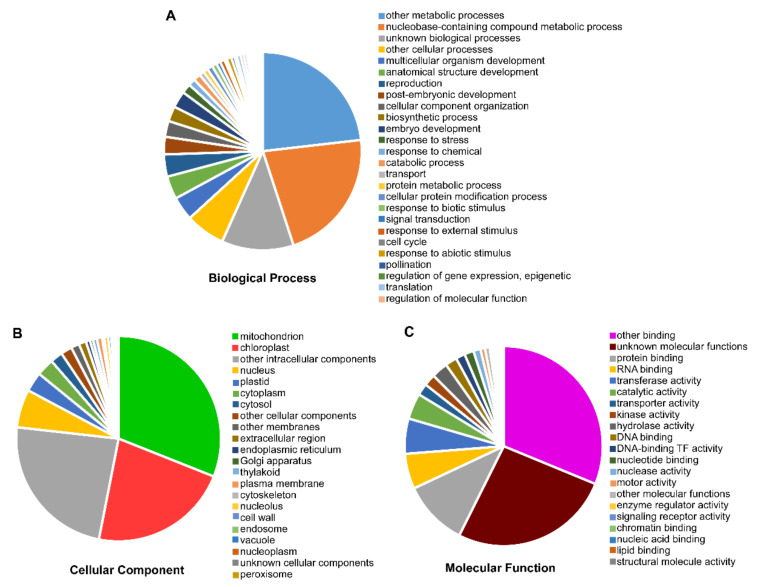
Functional annotation of ClaPPR proteins by Gene Ontology (GO) analysis. According to the GO annotation, the *ClaPPR* proteins were annotated into functional categories of (**A**) biological process, (**B**), cellular component, and (**C**) molecular function.

To elucidate the role of PPR genes in watermelon, GO annotations were performed for *ClaPPR*s. The results suggested that 364 of the 422 *ClaPPR* transcripts participated in biological processes (82.69%), cellular components (92.85%), and molecular functions (55.22%) (Figure 4). Further insights into the functional categorization indicated that a large portion of *ClaPPR*s were likely related to metabolic processes (179), followed by nucleobase-containing compound metabolic (168), unknown biological (91), and other cellular (50) processes (Figure 4A; Table S5). A total of 191 and 136 *ClaPPR* genes were found to be targeted to mitochondria and chloroplast, respectively; 146 to other intracellular components, 37 to nucleus, and 19 to plastids (Figure 4B; Table S5). For molecular functions, a total of 130 *ClaPPR* genes showed putative participation in binding functions such as protein (28), RNA (15), and DNA binding (5) and other bindings (82). We also found out that several *ClaPPRs* were predicted to be involved in activities, including transferase (15), catalytic (11), transporter (5), kinase (5), hydrolase (7), and unknown molecular functions (69) (Figure 4C; Table S5). In addition, GO enrichment analysis using AgriGO [34] also provided similar results as GO annotation. In the biological process category, all the *ClaPPR* families enriched for RNA modification (GO: 0009451) (Figure S4A). Among the molecular function, the binding functions such as zinc ion (GO: 0008270), translational metal ion (GO: 0043169), cation (GO: 0043169), and protein (GO: 0005515) binding were the enriched category (Figure S4B).

### 3.6. Expression Profiles of ClaPPR Genes in Different Stages of Fruit Development in 97103 Watermelon

**Figure 5:**
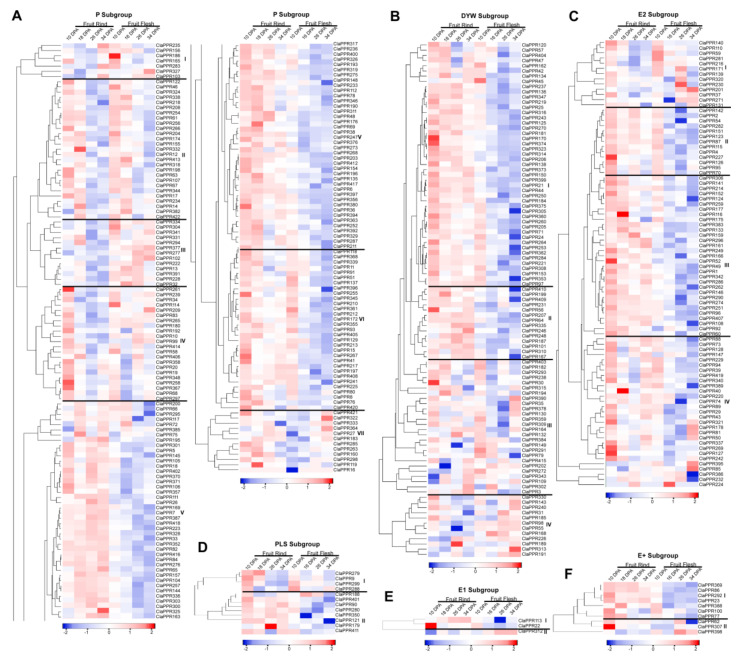
Expression profiles of *ClaPPR* genes during watermelon fruit development (Cultivar 97103; BioProject: SRP012849). All heat maps showing various expression levels of *ClaPPR* genes and subdivided into various clusters (labeled as roman numerals) were built using log2- transformed FPKM values of fruit rind and flesh at the developmental stages of 10, 18, 26, and 34 days after pollination (DAP). (**A**) Expression profiles of *ClaPPR* genes in the P subfamily, (**B**–**F**) Expression profiles of *ClaPPR* genes in the PLS subfamily and subgroups (**B**) DYW, (**C**) E2, (**D**) PLS, (**E**) E1, and (**F**) E+. Differences in transcript abundances such as high (red) and low (blue) levels are shown in color as the scale bar of *Z*-score.

Different PPR members may exhibit variations in the levels of mRNA accumulation among different tissues during the physiological processes of plants. To explore the putative biological functions of PPR members in watermelon, expression profiles of P- and PLS-subfamily members, including their subgroups (PLS, DYW, E1, E2, and E+), were investigated in fruit rind and flesh on different DAP during fruit development of watermelon 97103 using the RNA-seq data from the cucurbit expression atlas. Based on hierarchical clustering and expression heat map (Figure 5), *ClaPPR* genes from each subgroup were differentially expressed in rind and flesh parts during fruit development. However, the majority of the members from each subgroup exhibited preferential accumulation in rind compared to flesh and could therefore be clustered into different expression groups/clades. Based on the expression pattern, the P-subfamily members of 197 *ClaPPR* genes were distributed into seven distinct clades. Expression Clade-II of the P-subfamily includes a total of 27 genes displaying abundant expression at earlier DPAs (10 and 16 DPA) in both rind and flesh; however, expression levels declined at later stages (Figure 5A). The results suggest that these *ClaPPR* genes might be involved in the early stages of each tissue development (Figure 5A). Clade-III is comprised of 13 genes (*ClaPPR334*, *ClaPPR304*, *ClaPPR341*, *ClaPPR331*, *ClaPPR294*, *ClaPPR377*, *ClaPPR277*, *ClaPPR102*, *ClaPPR222*, *ClaPPR13*, *ClaPPR391*, *ClaPPR228*, and *ClaPPR32*), and was strongly upregulated in the flesh at almost all stages. Clade IV comprising 22 genes showed preferential accumulation in flesh; however, in the rind, the genes showed significant expression at only 10 DAP. Clade V, which is the largest one with 81 genes, displayed expression abundance only in the rind at almost all stages, suggesting that these genes could have a functional role in rind development. Clade VI and VII contain 31 and 11 genes (*ClaPPR421*, *322*, *333*, *364*, *27*, *183*, *285*, *263*, *160*, *298*, *119*, and *16*) with transcript accumulation majorly in the rind at all stages, however, their expression in the flesh was higher in early (10 DPA) and later (26 and 34 DPA) stages, respectively (Figure 5A).

Analysis of the expression patterns of other subgroups in the PLS subfamily indicated that clade I of DYW (47 genes), clade II (13 genes) and III (31 genes) of E2 and clade I of E+ (6 genes) of *ClaPPR* members showed significantly higher expression levels in the rind tissues of both stages compare to those in the flesh, where their expressions were only on early 10 DPA (Figure 5B). Similarly, in the DYW subgroup, some of the genes in clades II and III displayed upregulated expression patterns in the rind (10–34 DPA) and flesh (26–34 DPA). Flesh-specific expression of *ClaPPR* genes were also noted in clade IV of DYW (*ClaPPR330*, *143*, *240*, *31*, *185*, *98*, *55*, *168*, *226*, *189*, *313*, and *191*), where these genes were highly expressed at 26–34 DPA of flesh. Clade-I from the E2 subgroup, which comprises 13 genes, showed an up-regulated expression pattern in flesh tissues at 26 DPA (*ClaPPR281*, *216*, *171*, *139*, *320*, *230*, *201*, *37*, and *271*) and 34 DPA (*ClaPPR201*, *37*, *271*, and *131*) and displayed higher expressions in rind at 10 DPA (Figure 5C). Furthermore, clade IV of E2 (containing 29 genes) also possessed few genes (*ClaPPR321*, *178*, *81 50*, *242*, *395*, and *85*) that responded highly in flesh at 34 DPA. However, in the PLS-subgroup, a total of 7 genes (*ClaPPR401*, *90*, *280*, *350*, *121*, *179*, and *411*) were found to be more expressed in the rind than in the flesh at all DPA stages in clade II (Figure 5D). E1 and E+, which are smaller subgroups with only 3 and 10 *ClaPPR* members, respectively, showed a relatively higher expression in the rind (clade I of each) than in the flesh; however, only a few genes were expressed at a higher level in the flesh (*ClaPPR312* in clade of E1; *ClaPPR52*, *307*, and *395* in clade two E+) (Figure 5E,F). The result of the analysis indicates that *ClaPPR* genes in each subgroup showed high expression levels in both rind and flesh at all stages or particular stages of DPA; this facilitates the preliminary understanding of their possible participation in watermelon fruit development.

### 3.7. Comparative Expression Patterns of ClaPPR Genes under Different Fruit Ripening Stages of Two Cultivated Watermelon Varieties with Red- and Pale-Yellow-Fleshed

To explore the role of *PPR*s in fruit ripening of different flesh-colored watermelons, we investigated the comparative expression profiles of *ClaPPR* genes in fruit growth and ripening stages, including immature white (10 DPA), white-pink (18 DPA), red (26 DPA), and full-ripe (34 and 42 DPA), of two cultivated watermelons differing in flesh color, ‘COS’ (pale yellow-flesh) and ‘LSW-177’ (red-flesh) (Table S6) (BioProject: SRP012849). Almost all of the 422 *ClaPPR* genes showed expression in at least one of the DPA stages of the two watermelon varieties. It was noted that *ClaPPR* genes exhibited preferential and stage-specific expression between varieties. In the P-subfamily, most of the *ClaPPR* members showed a uniform upregulated expression in both COS and LSW177 at 10 DPA. However, at 26 DPA, these genes apparently exhibited abundant expression in LSW177 than in COS (Figure S5A). At 10 DPA, almost all of the *ClaPPR*s genes in both DYW and E2 subgroups showed significantly upregulated expression in the red flesh of LSW177 compared to that in COS. At 18 DPA, these genes exhibited robust, uniform upregulated expression patterns in both COS and LSW177, but relative to the remaining DPA stages (Figure S5B–C). The PLS subgroup members showed relatively similar expression levels in both COS and LSW177 at 10 DPA; and a higher level in LSW177 than in COS at 26 DPA as observed in the P-subgroup (Figure S5D). Both E1 and E+ subgroup members exhibited significant expression in LSW117 than inCOS at 10 DPA, while their expression at 18 DPA were almost uniform between LSW117 and COS as observed in DYW and E2 subgroups (Figure S5E–F). At the full-ripe stages (34 and 42 DPA), only the P-subfamily exhibited significant expression; some of the *ClaPPR* members had high transcript accumulation in COS than in LSW117, particularly at 34 DPA (Figure S5A and D). These results suggest that *ClaPPR* genes can be considered as candidate genes that are associated with growth and ripening of watermelon fruits.

### 3.8. Sequence Variation in ClaPPR Genes and Development of CAPS Markers for Flesh Color

**Figure 6:**
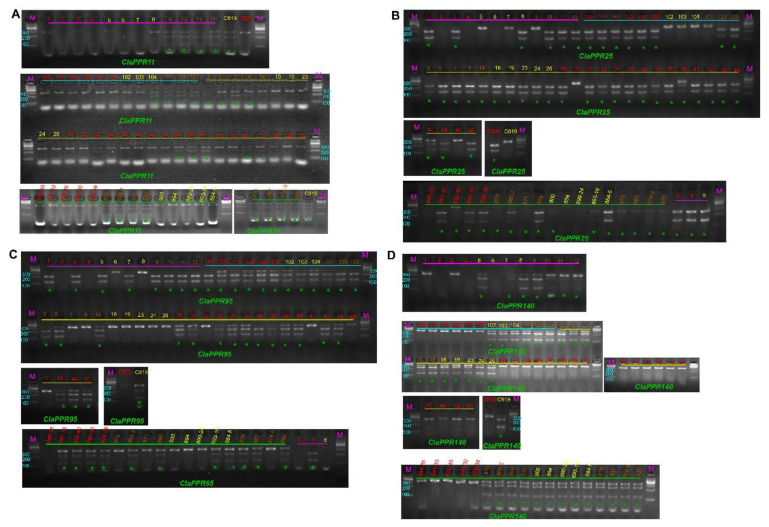
Gel pictures for the validated cleaved amplified polymorphic sequence (CAPS) markers based on single-nucleotide polymorphisms (SNPs) in *PPR* genes. The name of validated CAPS markers such as *ClaPPR11* (**A**), *ClaPPR25* (**B**), *ClaPPR95* (**C**), and *ClaPPR140* (**D**) are shown below their corresponding gel pictures. Numbers in lanes underlined with different colors indicates the DNA sample names of the 70 lines, same as those listed in Table S7. M represents a 100 bp ladder. A “*green asterisk*” represents the enzyme-cleavage of the DNA samples. DNA samples of C803 (red-flesh) and C819 (yellow-flesh) were used as control (Table S7) during the restriction digestion of PCR amplicons.

For the utilization of *ClaPPR* genes in watermelon breeding, we investigated the relationship between the watermelon flesh color and sequence variations in *ClaPPR* genes. We identified the SNPs in the sequences of *ClaPPR* encoding genes from 24 different flesh colored watermelons (red, yellow, and orange) using our WGRS data (Bioproject: PRJNA516776) [29]. A total of 368 SNPs were observed from 139 *ClaPPR* genes in the WGRS data. After detailed analysis of all SNPs, we selected 4 SNP-carrying genes, including *ClaPPR11*, *ClaPPR25*, *ClaPPR95*, and *ClaPPR140* from 9 red, 9 yellow, and 6 orange flesh-colored watermelons, and compared them with the reference 97103 genome (Table S7). The SNPs in *ClaPPR* genes were found to be monomorphic among a chosen flesh color-type and polymorphic between a chosen and an unchosen flesh color-types; each SNP in the corresponding *ClaPPR* gene almost showed a co-segregation with a particular flesh color phenotype (Orange: *ClaPPR11*, Yellow: *ClaPPR25* and *ClaPPR95*, and Red: *ClaPPR140*). Among the four SNP-carrying genes, 3 of them were classified as non-synonymous substitutions (*ClaPPR11*, *ClaPPR25*, and *ClaPPR140*) with altered amino acid residue in the PPR motifs, which could cause functional variation, in those corresponding genes, between a desired and non-desired flesh types. Therefore, to detect the association among the four SNP-carrying *ClaPPR* genes and flesh colors, CAPS marker primer sets were designed and analyzed by restriction digestion. Using these four CAPS markers, genotyping was carried out on 70 different commercial cultivars comprising red (33), yellow (17), and orange (20) flesh color for their reliability and applicability on watermelon breeding (Figure 6; Table S7). The genotyping results for flesh color determination, based on the SNPs of the four *ClaPPR* genes, are described in Table S8. *ClaPPR11* showed a higher match rate of 0.87 (87%) among genotypes of the markers and phenotypes of the orange-flesh color in all surveyed lines. However, *ClaPPR140* had a perfect co-segregation with red-flesh color with a match rate of 1 (100%). With regard to genotyping for *ClaPPR25* and *ClaPPR95*, they co-segregated well with yellow-flesh, exhibiting a match rate of 0.79 and 0.76, respectively (Table S8). Furthermore, a joint, *ClaPPR25* + *ClaPPR95* genotyping provided an average match rate of 0.94, indicating a high reliability and applicability of flesh type specific *ClaPPR* gene-based SNPs identified in this study.

## 4. Discussion

Watermelon (*Citrullus lanatus*) is one of the important economic crops in the *Cucurbitaceae* family. Fruits of watermelon contain sugars, carotenoids (lycopene, beta-carotene, and phytoene), and various health-promoting nutritional compounds (glutathione, citrulline, and arginine) which significantly contributes to the human diet [28]. With a relatively compact genetic complement (~425 Mbp), the gene families of watermelon are being investigated owing to their sequenced or re-sequenced draft genomes [29,42]. PPR proteins are one of the largest gene families in terrestrial plants. In the present study, 422 PPR proteins, of which 197 and 225 members belonged to the P subfamily and PLS subfamily, respectively, were identified in the 97103 watermelon genome (Figure 1; Table S2). This number of proteins is in accordance with previous studies that reported the presence of >400 PPR genes per plant genome, including *Arabidopsis*, foxtail millet, maize, and rice [1,5,6,7]. Analysis of gene structure revealed that more than 70% of the *ClaPPR* genes were intronless, indicating that a majority of the plant PPR genes (for example, 80% of *PPR* genes in Arabidopsis) lack introns [3,5,7], as it could be the result of amplification by retrotransposition events, in which intron-poor genes might have originated from intron-rich PPRs [1,43,44].

Although analysis of conserved protein motif analyses did not reveal the same results as that for the motifs used to distinguish different types of PPR proteins, it can be used to determine the conserved molecular functions in P and PLS subfamilies of PPR genes [6]. Conserved motif analysis showed that 25 motifs are present in *ClaPPR* proteins; motif 21 and 25 are present only in the P subfamily; the PLS subfamily of the DYW subgroup only exhibits motif 5, 13, and 20; and both DYW and E2 share motifs 3, 7, and 17 (Figure S2). This type of motif distribution has also been observed for PPR proteins in maize where some motifs were even conserved between their genomes [6]. These identified subgroup-specific motifs could be a significant component of the corresponding *ClaPPR* genes in different subgroups that may determine the conserved molecular functions. However, extensive future studies on the characterization of these *ClaPPRs* are required to elucidate their conserved functions. The *ClPPR*s were unevenly distributed on each chromosome, and often clustered together in short regions of the chromosomes (Figure 1A and Figure 2). These results indicated that the size of chromosomes was not relatively associated with the number of genes [31] and that duplication events could have resulted in the expansion of these genes as suggested in a previous study [5]. In duplicated *ClPPR* gene analysis, we noted a total of 11 segmentally duplicated genes located on each of all the chromosomes (Figure 2; Table S3), suggesting that segmental duplication is the most prevalent, having a higher frequency than tandem duplication events in watermelon, and correspond to that reported in previous genome-wide studies [45,46]. In our phylogenetic analysis, *ClaPPR* proteins, based on the pattern of PPR motifs, can be classified into two groups of proteins: P subfamily and PLS subfamily (Figure 3). It has been observed that several P subfamily proteins clustered together with PLS subfamily proteins, showing similarity in the evolutionary relationship of PPR proteins in poplar and rice [3,7].

PPR proteins have been predominantly predicted to be located in the mitochondria and plastids [7]. In the present study, most of the identified *ClaPPR* proteins (approximately 65%) were predicted to be commonly localized in subcellular regions of the chloroplast (73 of 422) and mitochondria (204 of 422) (Figure S3). GO analysis also indicated that many *ClaPPR* proteins seem to be located in the mitochondria (191) and chloroplast (136) (Figure 4). PPR proteins modulate gene expression via post-transcriptional or translational regulations in organelles at the RNA level by acting as RNA-binding proteins [47]. It has also been observed that a large number of *ClPPRs* had binding functions, including DNA, RNA, and protein binding and other-binding functions (Figure 4 and Figure S4), corroborating the results of PPR protein functions. Therefore, any defects or mutations in organelle-targeted PPR proteins often result in organelle dysfunction, which ultimately leads to altered phenotypes, including cytoplasmic male sterility [24], defective embryo development [12], abnormal photosynthetic ability and aberrant pigmentation in seeds [48], and flesh color variation [27]. Future studies on the functional characterization of *ClaPPR* genes will clarify their significant implications in watermelon breeding.

There is increasing evidence that PPR genes play a significant role in plant growth and developmental process, and their mRNA expression patterns have been explored in cotton floral buds [31], maize kernels [6], and rice panicles [7]. In the present study, we investigated the expression patterns of *ClaPPR* genes in watermelon fruit development (BioProject: SRP012849). The results of in-silico expression analyses indicated that *ClaPPRs* were differentially expressed in the rind and flesh tissues (Figure 5). Watermelon fruits have rapid cell division and expansion in their early fruit development stages, resulting in changes in cell wall structure and accumulation of compounds (carbohydrates and organic acids) in vacuoles; followed by fruit ripening stages, which involves changes in carotenoid profiles and conversion of carbohydrates to sugars [49]. The expression levels of some of the *ClaPPR* members in the P-subfamily were relatively high in fruit flesh (Clade III), or fruit rind (Clade V), and or both flesh and rind tissues (Clade II, IV, VI, and VII), suggesting that they could be important in correlating the development of rind and flesh in watermelon (Figure 5A). In the PLS-subfamily, most of the subgroups were preferentially expressed in rind with a higher level at all DPA as well as flesh at 10 DPA (clade I of DYW; clade II, III of E2; and clade I of E+), suggesting that these genes might be important in fruit rind and early fruit flesh development (Figure 5B–F). DYW (clade II, III, and IV) and E2 (clade IV) members presented high expression levels in the fruit flesh at the late ripening stages (26 and 34 DAP), indicating that these genes might be essential for carotenoid accumulation and fruit ripening of watermelon (Figure 5B–C). These results imply that *ClaPPR* genes might be involved in watermelon fruit development and chloroplast-to-chromoplast transition.

Fruit flesh color is an important trait of watermelon; variations in carotenoid profiles often result in colors of red (lycopene), yellow (phytoene), and orange (β-carotene) fleshes [29]. Fruit ripening has been reported to be influenced by environmental factors, hormones, and developmental gene regulation [49,50]. Therefore, identification and characterization of genes, which govern fruit growth and ripening, would be helpful in watermelon breeding. The present study explored the expression profiles of *ClaPPR* genes to evaluate the possible roles of *ClaPPR* in fruit growth and ripening stages (10–42 DPA), between ‘COS’ (pale yellow-flesh) and ‘LSW-177’ (red-flesh) watermelons (BioProject: PRJNA338036). At 10 and 18 DPA, *ClaPPR* genes from various subgroups were found to be generally upregulated in both COS and LSW177 (Figure S5). However, DYW, E2, and E+ subgroup genes displayed robust upregulated expression of their transcripts in LSW177 than in COS. Similarly, both P- and PLS- subgroups were also upregulated only in LSW177 at 26 DPA. In contrast, these two subgroups showed high transcript accumulation in COS than in LSW117 at full-ripening stages of 34 DPA (Figure S5A,D). From the expression profiles, it was also speculated that the expression of 242 and 226 *ClaPPR* genes were upregulated at all stages in LSW177 and COS, respectively, among which 60 and 52 genes showed high expression levels (a log2 value between 3–5) (Figure S5; Table S6). This indicated that LSW177 had 1.07 (242/226 = 1.07) fold higher number of upregulated *ClaPPR* genes than that of COS. Therefore, it appears that the mechanism of fruit growth and ripening in LSW177 is more complex than that in COS, and that the PPR family also has functional involvement in the growth and ripening of fruits. However, further studies are required to elucidate the complete role of these upregulated *ClaPPR*s in watermelon fruit.

mRNA expression of PPR genes have been reported to be regulated by microRNAs (miRNA) through cleavage or translational repression in plants [3,7,51]. In watermelon, a previous study showed that eight PPR genes (Cla008388, Cla012681, Cla015802, Cla018752, Cla011015, Cla005585, Cla019381, and Cla006187) have complementary sites of miRNAs [52]. These miRNAs have been reported to be involved in melatonin-mediated cold tolerance in watermelon by suppressing the expression of the abovementioned PPR genes through either cleavage (gene names as in the present study; *ClaPPR29*:*miR399-5p*, *ClaPPR268*:*miR8029-39*, and *ClaPPR59*:*novel-m0058-5p*), or translational inhibition (*ClaPPR234*:*miR159-5p*, *ClaPPR21*:*miR6284-3p*, *ClaPPR348*:*novel-m0030-5p*, *ClaPPR104*:*novel-m0051-5p*, and *ClaPPR179*:*novel-m0051-5p*) [52]. In addition, a recent study showed that a total of 218 PPR genes have complementary sites of 160 miRNAs [53]. Hence, further research is required on the dynamic expression of miRNAs and their corresponding *ClaPPR* targets, and a crosstalk between miRNAs and PPRs will contribute to the regulation of plant growth and fruit development in watermelon.

Based on the SNPs in *ClaPPR* genes (Table S7), the developed four CAPS were found to perfectly co-segregated with their corresponding flesh colors, and match rate ranged from 0.87 to 1 (Figure 6; Table S8). Notably, *ClaPPR11* co-localized with β-carotene-related QTL on chromosome Chr1 [54], whereas *ClaPPR140* co-localized with lycopene-related QTL on chromosome Chr4 [55]. With regard to *ClaPPR25* and *ClaPPR95*, they were not co-localized with any QTL for flesh color. Therefore, the identified SNPs in these *ClaPPR* genes might be used for fine mapping of flesh color locus in watermelon genome. Few amino acid positions (4th and 34th) in a PPR motif have been found to act as attachment points, which help PPR proteins to binds to target mRNAs, thus inhibiting translation [56]. In Arabidopsis, a single nonsynonymous mutation at the 4th amino acid in the 12th PPR motif inhibited the complete function of a PPR gene called *Proton Gradient Regulation3* [57]. Among the selected SNP-carrying candidate genes, *ClaPPR11* and *ClaPPR140* had nonsynonymous mutation at the 2nd amino acid location in the 13th and 11th motif, respectively, while *ClaPPR25* had nonsynonymous mutation at the 23rd amino acid location in the 18th motif (Table S7), suggesting that these SNPs could influence the binding action of corresponding *ClaPPR* and therefore, play a role in their functions. Furthermore, the aforementioned *ClaPPR* protein-encoding genes could be considered as important candidates for watermelon fruit related traits and the developed CAPS markers will be helpful for breeders to economically distinguish fruit flesh colors at watermelon seedling stage.

## 5. Conclusions

In this study, a total of 422 PPR protein genes were identified in the watermelon genome. Based on the PPR motif type, *ClaPPR* genes were divided into five subgroups. Most of the genes were intronless and distributed widely across all watermelon chromosomes, and encoding proteins were targeted to organelles of chloroplast or mitochondria which gives valuable information for future studies on characterization of *ClaPPR* genes. Duplication analyses suggested that 11 segmentally duplicated *ClaPPR* pairs exist in the genome. We conducted an in-silico expression pattern analysis in watermelon fruit rind and flesh tissues. In addition, a comparative expression study was performed in the fruit ripening stages of red- and pale yellow-fleshed watermelons, which provides preliminary understanding about *ClaPPR* participation in fruit development. Based on sequence variation analyses of *ClaPPR* genes, four CAPS markers were developed and found to have co-segregation with distinct flesh types, which could be used to distinguish different flesh colors, including red, yellow, and orange. Taken together, the findings of this study provide comprehensive understanding of the *ClaPPR* gene family and clarify candidate *ClaPPR* genes for functional validation in the future.

## Supplementary Materials

The following are available online at https://www.mdpi.com/2073-4425/11/10/1125/s1, Figure S1. Conserved motifs in *ClaPP*R proteins using MEME Suite, Figure S2. Conserved motifs of *ClaPPR* proteins. The consensus sequence of motif logos shown in the left panel. The number of motifs in the different subfamilies and/or subgroups of the *ClaPPR* proteins are shown in the right panel, Figure S3. Subcellular localizations of *ClaPPR* genes, using on-line tools of TargetP 2.0 and Predotarv.1.04. The locations of each *ClaPPR* subfamily and its subgroups in the chloroplast (blue), mitochondria (orange), endoplasmic reticulum (ER) (grey), and other locations (yellow) were determined, Figure S4. GO enrichment analysis of *ClaPPR* genes. (A) Graphical results of biological process. (B) Graphical results of molecular function, Figure S5. A comparative expression profiles of *ClaPPR* genes during watermelon fruit development between red-fleshed (LSW177) and pale-yellow-fleshed cultivated watermelons (BioProject: PRJNA338036). The heat map with clustering (labeled as roman numerals) was generated based on the log2- transformed FPKM value of *ClaPPR* genes at developmental stages of 10, 18, 26, 34, and 42 days after pollination (DAP). (A) Expression profiles of *ClaPPR* genes in the P subfamily, (B–F) Expression profiles of *ClaPPR* genes in the PLS subfamily of subgroups (B) DYW, (C) E2, (D) PLS, (E) E1, and (F) E+. Differences in transcript abundances such as high (red) and low (blue) levels are shown in color as the scale, Table S1. List of watermelon commercial cultivars or inbred lines used in this study for CAPS marker validation and their representative fruit characteristics, Table S2. Detailed information of PPR genes in watermelon genome, Table S3. Segment duplication of PPR genes in watermelon genome, Table S4. Subcellular localization of predicted PPR genes in watermelon, Table S5. Details of GO annotation of *ClaPPR* genes, Table S6. Log2 FPKM values for *ClaPPR* genes in different fruit ripening stages of two cultivated watermelon varieties with red-fleshed (LSW-177) and pale-yellow-fleshed (COS), Table S7. Identification of putative sequence variation (SNPs) between lines with flesh-colored (red, yellow, and orange) watermelons from the WGRS data, Table S8. Summary of result analysis of match rate between validated *ClaPPR* CAPS markers and SNPs in 70 watermelons.
