# Supplementary material for: A Genome-Wide Analysis of the Pentatricopeptide Repeat (PPR) Gene Family and PPR-Derived Markers for Flesh Color in Watermelon (Citrullus lanatus)
Source: Genes (Basel). 2020 Sep 24;11(10):1125. doi: 10.3390/genes11101125 (PMC7650700; doi:10.3390/genes11101125)
Supplement: Supplementary file 1 [file genes-11-01125-s001.zip › genes_925536-Supplementary/Supplemtary Figures/Figure S1 .pdf]

Figure S1. The Conserved motifs in ClaPPR proteins by using MEME Suite.

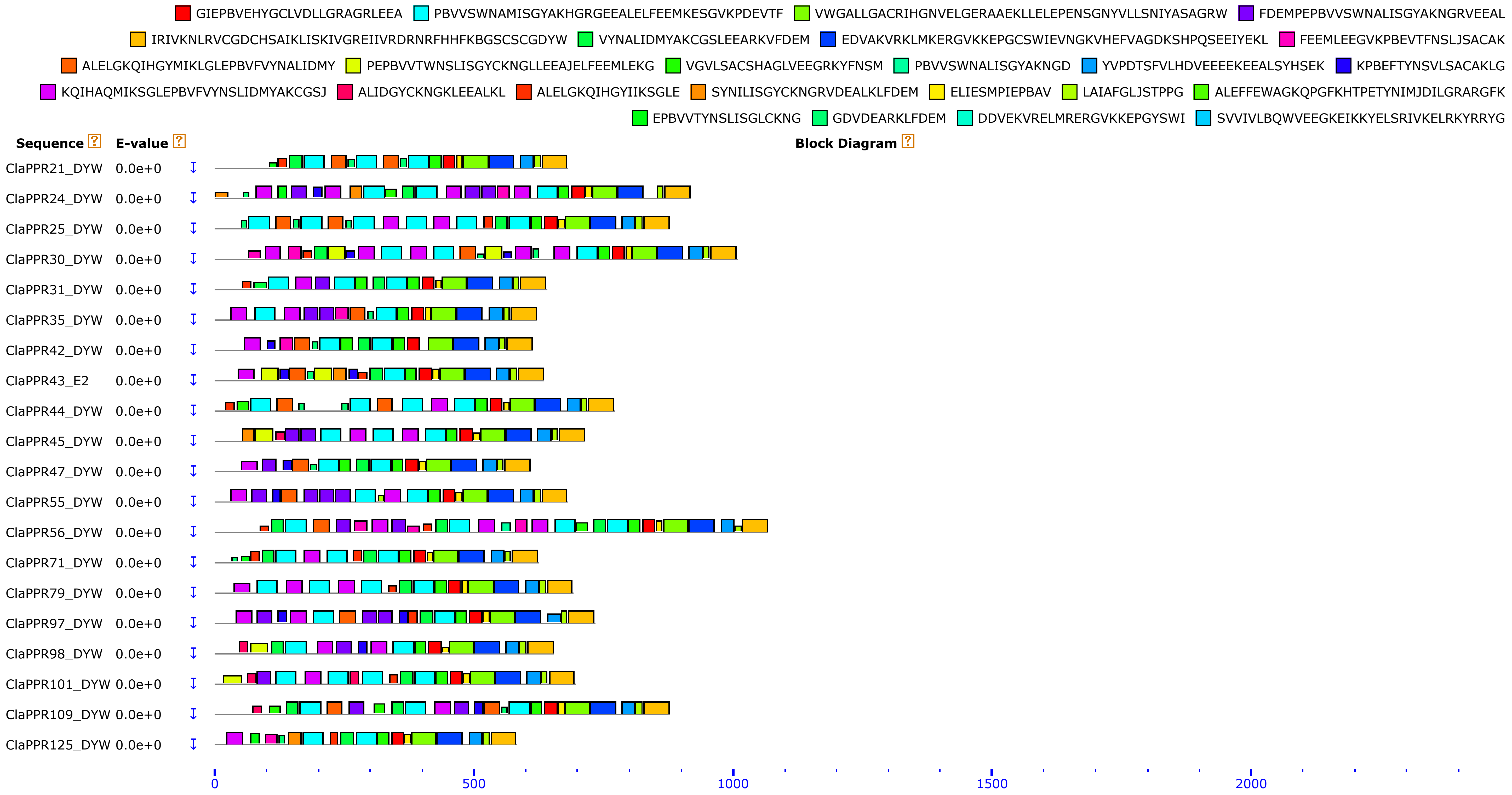

Sequence	E-value
ClaPPR130_DYW	0.0e+0
ClaPPR132_DYW	0.0e+0
ClaPPR134_DYW	0.0e+0
ClaPPR136_DYW	0.0e+0
ClaPPR138_DYW	0.0e+0
ClaPPR143_DYW	0.0e+0
ClaPPR150_DYW	0.0e+0
ClaPPR153_DYW	0.0e+0
ClaPPR162_DYW	0.0e+0
ClaPPR164_DYW	0.0e+0
ClaPPR168_DYW	0.0e+0
ClaPPR170_DYW	0.0e+0
ClaPPR182_DYW	0.0e+0
ClaPPR184_DYW	0.0e+0
ClaPPR185_DYW	0.0e+0
ClaPPR187_DYW	0.0e+0
ClaPPR189_DYW	0.0e+0
ClaPPR191_DYW	0.0e+0
ClaPPR194_DYW	0.0e+0
ClaPPR199_DYW	0.0e+0
ClaPPR202_DYW	0.0e+0
ClaPPR205_DYW	0.0e+0
ClaPPR206_DYW	0.0e+0
ClaPPR221_DYW	0.0e+0
ClaPPR226_DYW	0.0e+0
ClaPPR231_DYW	0.0e+0
ClaPPR237_DYW	0.0e+0
ClaPPR238_DYW	0.0e+0
ClaPPR240_DYW	0.0e+0
ClaPPR243_DYW	0.0e+0
ClaPPR246_DYW	0.0e+0
ClaPPR250_DYW	0.0e+0
ClaPPR253_DYW	0.0e+0
ClaPPR260_DYW	0.0e+0

Block Diagram

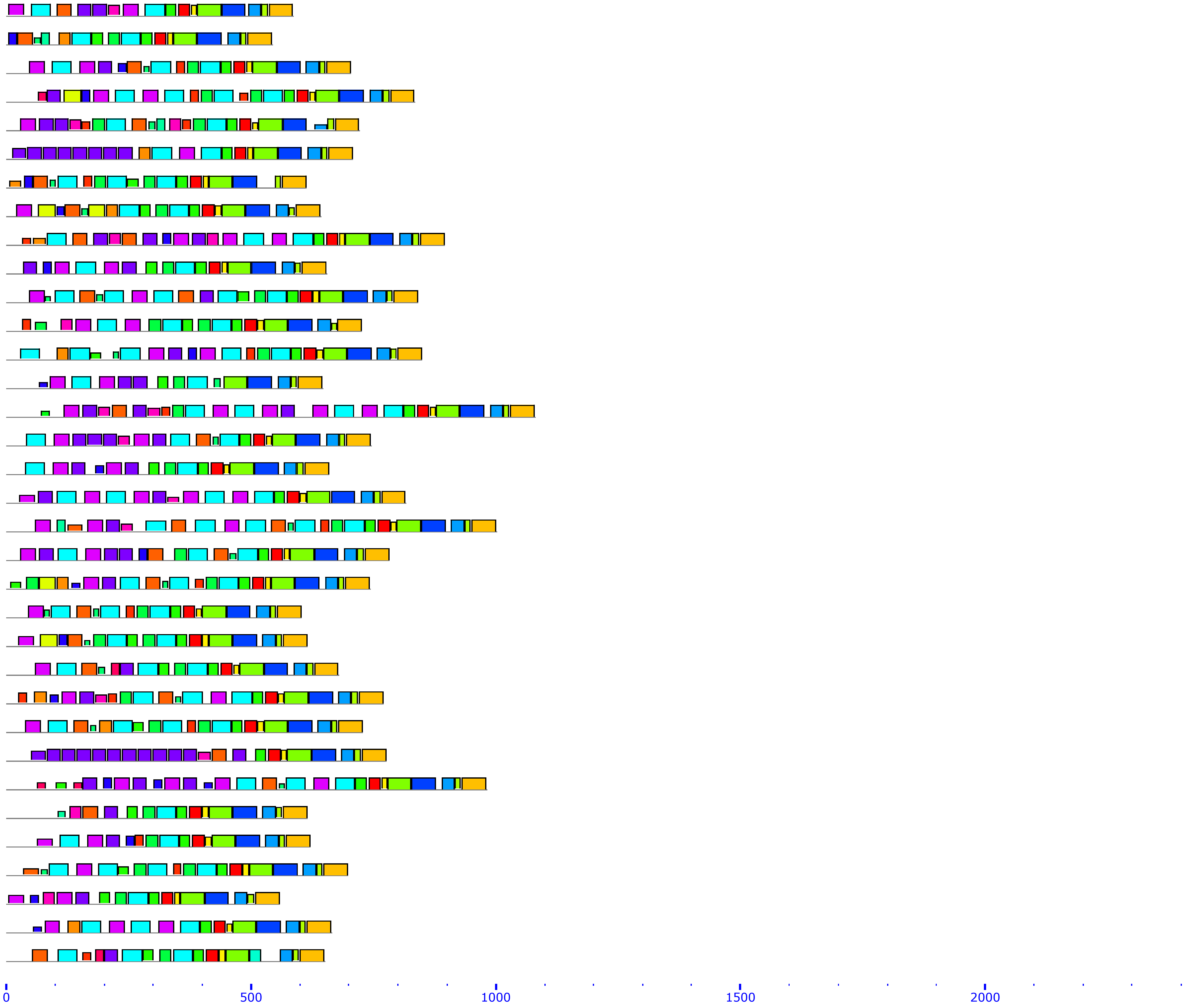

Sequence	E-value
ClaPPR264_DYW	0.0e+0
ClaPPR272_DYW	0.0e+0
ClaPPR284_DYW	0.0e+0
ClaPPR291_DYW	0.0e+0
ClaPPR293_DYW	0.0e+0
ClaPPR305_DYW	0.0e+0
ClaPPR308_DYW	0.0e+0
ClaPPR309_DYW	0.0e+0
ClaPPR313_DYW	0.0e+0
ClaPPR314_DYW	0.0e+0
ClaPPR315_DYW	0.0e+0
ClaPPR316_DYW	0.0e+0
ClaPPR323_DYW	0.0e+0
ClaPPR330_DYW	0.0e+0
ClaPPR335_DYW	0.0e+0
ClaPPR343_DYW	0.0e+0
ClaPPR347_DYW	0.0e+0
ClaPPR353_DYW	0.0e+0
ClaPPR359_DYW	0.0e+0
ClaPPR360_DYW	0.0e+0
ClaPPR373_DYW	0.0e+0
ClaPPR374_DYW	0.0e+0
ClaPPR375_DYW	0.0e+0
ClaPPR378_DYW	0.0e+0
ClaPPR384_DYW	0.0e+0
ClaPPR390_DYW	0.0e+0
ClaPPR399_DYW	0.0e+0
ClaPPR403_DYW	0.0e+0
ClaPPR404_DYW	0.0e+0
ClaPPR409_DYW	0.0e+0
ClaPPR410_DYW	0.0e+0
ClaPPR415_DYW	0.0e+0
ClaPPR292_E+	8.4e-274
ClaPPR3_DYW	1.3e-269

Block Diagram

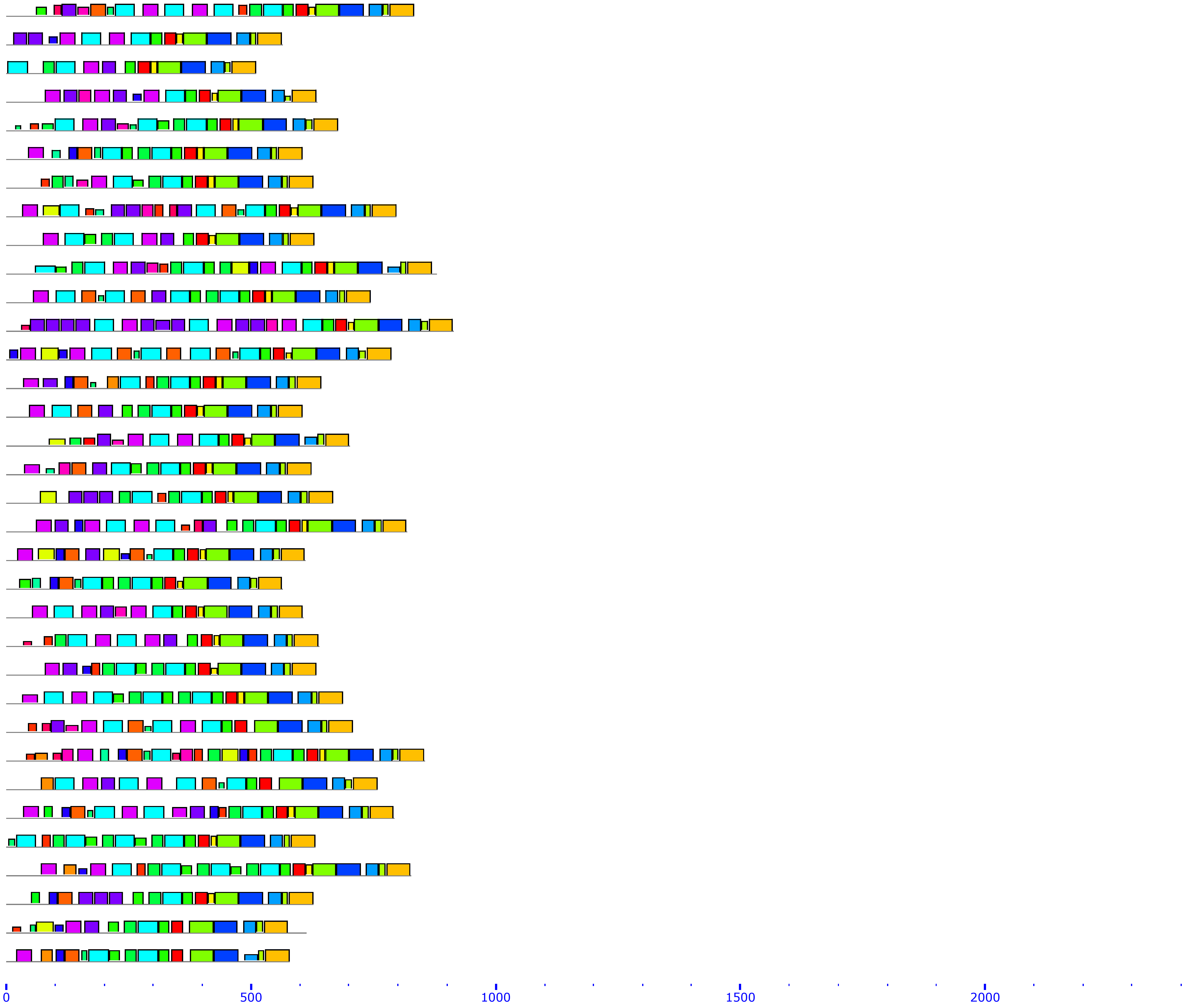

Sequence	E-value
ClaPPR302_DYW	1.3e-267
ClaPPR278_DYW	6.2e-267
ClaPPR248_DYW	1.7e-261
ClaPPR207_DYW	8.6e-260
ClaPPR388_E+	1.5e-259
ClaPPR369_E+	8.6e-257
ClaPPR124_E2	2.1e-256
ClaPPR230_E2	2.5e-256
ClaPPR219_DYW	5.9e-256
ClaPPR372_E2	1.2e-255
ClaPPR159_E2	1.6e-254
ClaPPR321_E2	9.3e-254
ClaPPR227_E2	2.9e-253
ClaPPR149_DYW	3.3e-253
ClaPPR307_E+	1.1e-251
ClaPPR140_E2	5.4e-250
ClaPPR62_E+	3.2e-248
ClaPPR23_E+	4.4e-247
ClaPPR95_E2	9.2e-244
ClaPPR282_E2	1.6e-243
ClaPPR126_E2	4.8e-242
ClaPPR177_E2	5.4e-242
ClaPPR407_E2	2.2e-240
ClaPPR381_E2	8.2e-240
ClaPPR310_DYW	3.0e-239
ClaPPR419_E2	3.6e-239
ClaPPR337_E2	4.9e-239
ClaPPR398_E+	1.2e-238
ClaPPR161_E2	3.7e-236
ClaPPR181_DYW	5.3e-236
ClaPPR86_E+	1.2e-235
ClaPPR251_E2	3.9e-235
ClaPPR383_E2	3.0e-234
ClaPPR166_E2	9.2e-234

Block Diagram

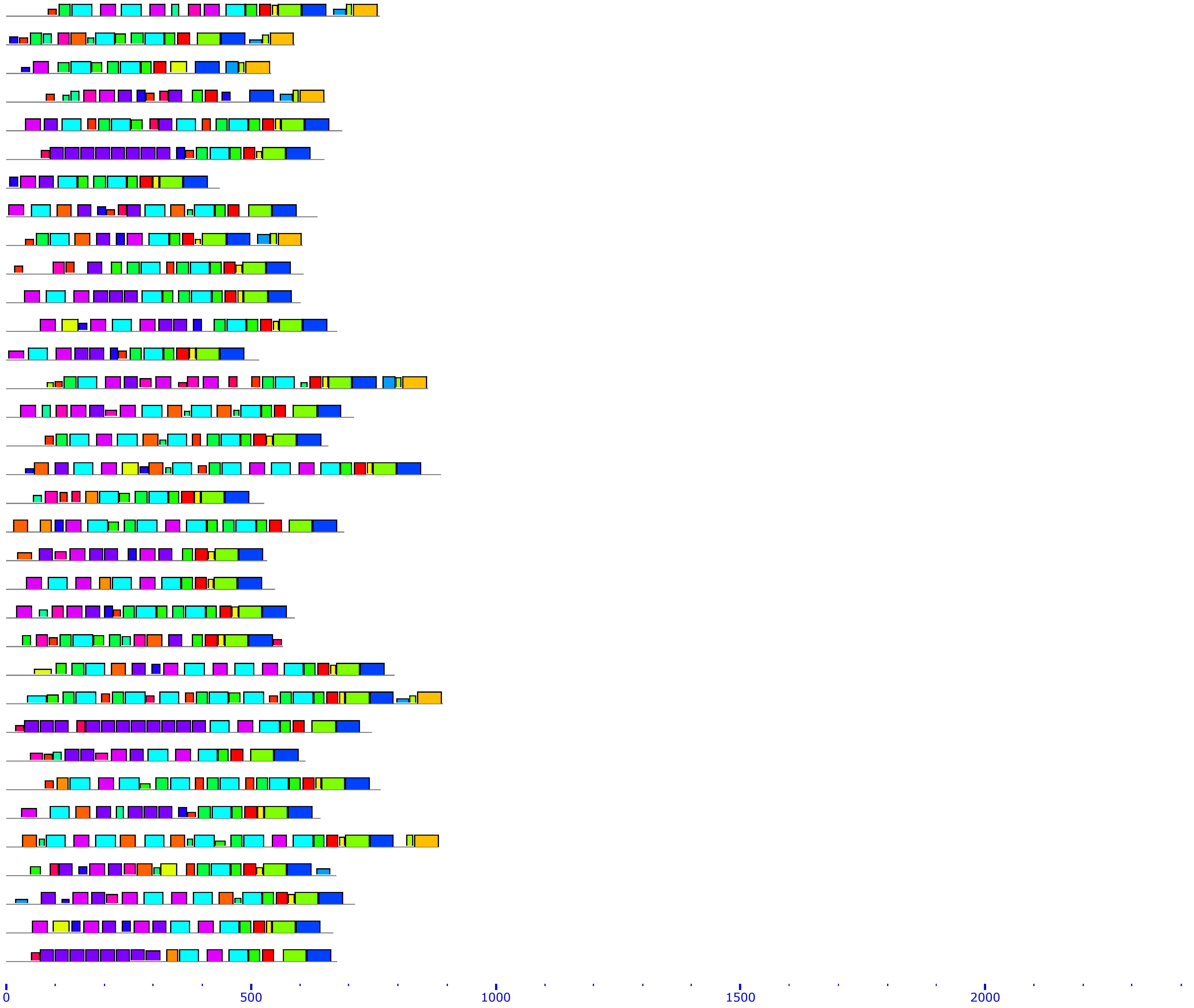

Sequence	E-value
ClaPPR146_E2	2.8e-233
ClaPPR96_E2	3.0e-233
ClaPPR178_E2	3.2e-233
ClaPPR94_E2	8.2e-233
ClaPPR167_DYW	1.1e-232
ClaPPR274_E2	1.8e-232
ClaPPR171_E2	3.7e-232
ClaPPR286_E2	5.4e-232
ClaPPR147_E2	2.8e-231
ClaPPR262_E2	8.1e-231
ClaPPR60_E2	8.5e-229
ClaPPR229_E2	3.8e-228
ClaPPR281_E2	3.8e-227
ClaPPR108_E2	4.2e-227
ClaPPR77_E+	3.4e-226
ClaPPR269_E2	6.2e-226
ClaPPR271_E2	1.9e-225
ClaPPR115_E2	3.0e-225
ClaPPR249_E2	7.2e-225
ClaPPR133_E2	1.2e-223
ClaPPR1_E2	1.8e-223
ClaPPR52_E2	2.6e-223
ClaPPR88_E2	2.5e-222
ClaPPR362_DYW	1.3e-220
ClaPPR365_E2	2.7e-220
ClaPPR306_E2	6.4e-219
ClaPPR351_E2	8.1e-219
ClaPPR74_E2	3.4e-217
ClaPPR379_E2	5.1e-217
ClaPPR342_E2	1.3e-216
ClaPPR128_E2	3.6e-216
ClaPPR389_E2	1.6e-215
ClaPPR59_E2	7.6e-215
ClaPPR220_E2	2.3e-214

Block Diagram

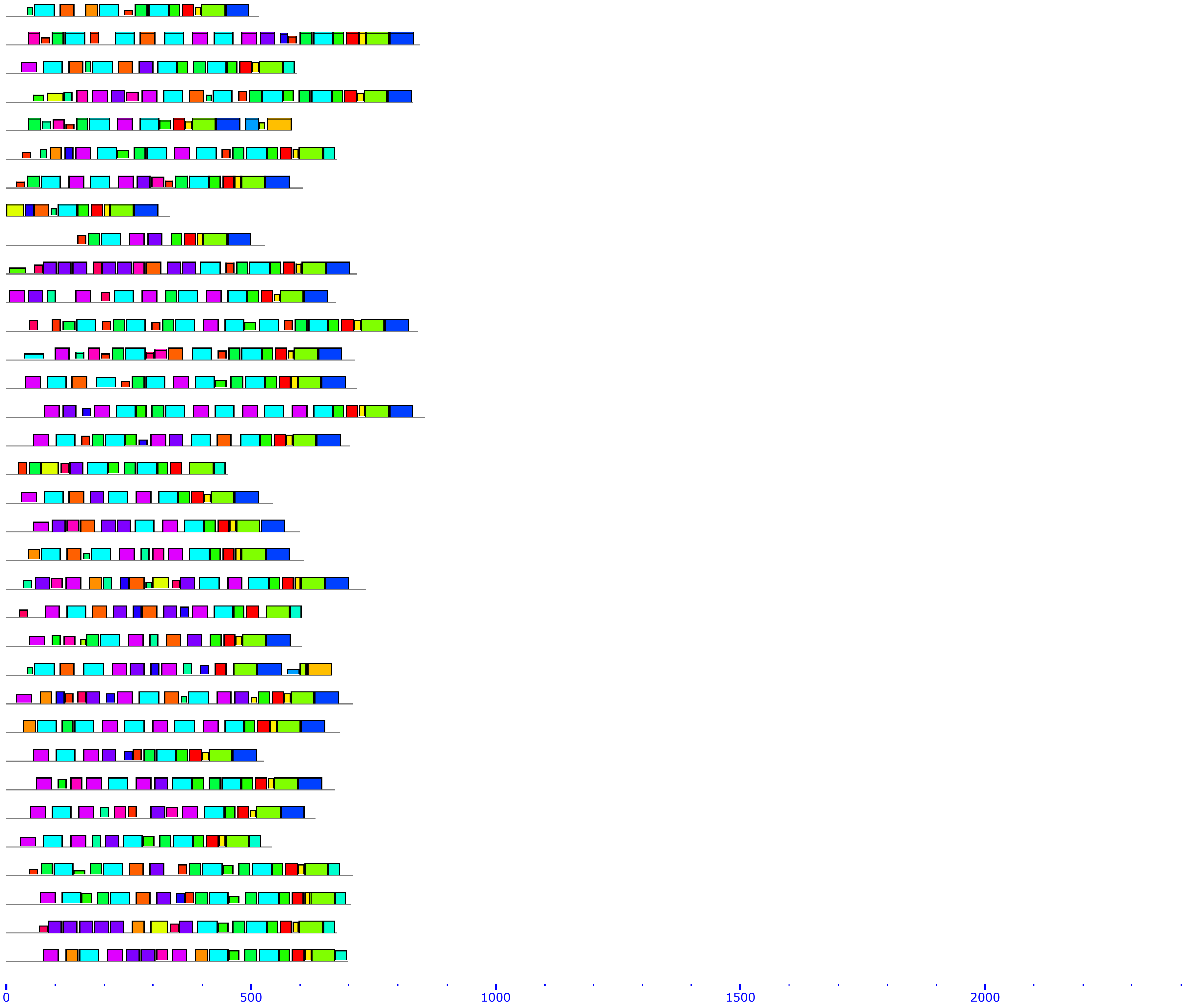

Sequence	E-value
ClaPPR296_E2	1.1e-211
ClaPPR50_E2	1.3e-211
ClaPPR151_E2	2.0e-211
ClaPPR70_E2	2.9e-211
ClaPPR100_E+	2.4e-210
ClaPPR29_E2	4.3e-209
ClaPPR92_E2	4.3e-203
ClaPPR39_E2	1.1e-202
ClaPPR85_E2	1.2e-202
ClaPPR232_E2	2.2e-202
ClaPPR123_E2	1.2e-201
ClaPPR175_E2	1.3e-201
ClaPPR40_E2	1.6e-201
ClaPPR201_E2	5.2e-201
ClaPPR270_DYW	2.2e-200
ClaPPR142_E2	6.5e-200
ClaPPR366_E2	1.0e-199
ClaPPR131_E2	1.7e-199
ClaPPR349_E2	2.9e-199
ClaPPR141_E2	9.6e-199
ClaPPR4_E2	1.0e-198
ClaPPR242_E2	1.5e-198
ClaPPR28_E2	2.2e-195
ClaPPR87_E2	2.4e-195
ClaPPR340_E2	1.0e-190
ClaPPR2_E2	1.9e-190
ClaPPR152_E2	2.1e-190
ClaPPR116_E2	1.1e-189
ClaPPR386_E2	3.0e-188
ClaPPR216_E2	1.2e-187
ClaPPR320_E2	2.2e-187
ClaPPR120_DYW	6.5e-186
ClaPPR12_P	6.1e-185
ClaPPR8_P	4.0e-184

Block Diagram

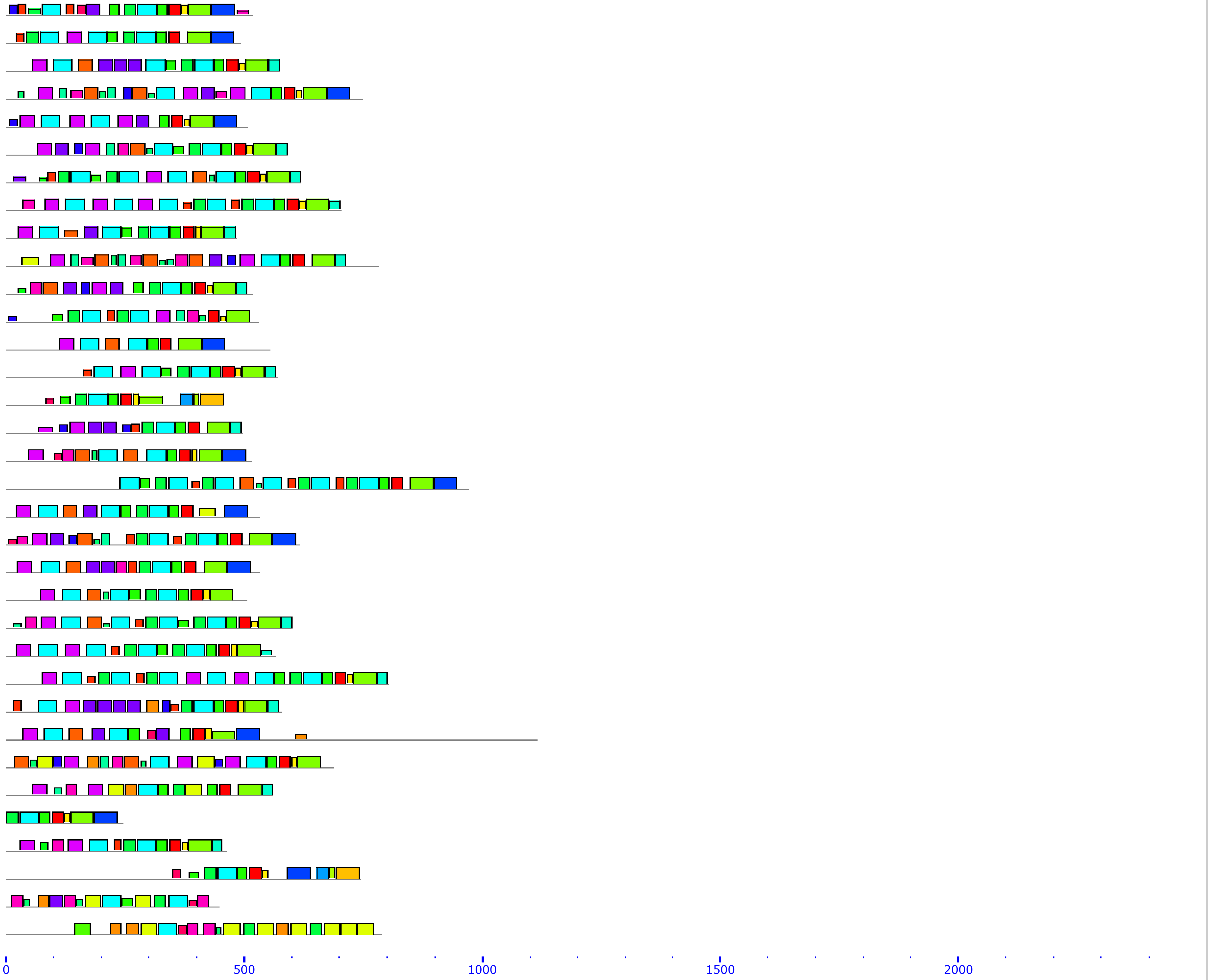

Sequence	E-value
ClaPPR89_E2	2.8e-182
ClaPPR139_E2	3.9e-182
ClaPPR49_E2	9.8e-182
ClaPPR214_E2	3.7e-181
ClaPPR259_E2	5.0e-181
ClaPPR110_E2	4.6e-179
ClaPPR127_E2	1.3e-178
ClaPPR73_E2	2.9e-178
ClaPPR354_E2	1.3e-177
ClaPPR290_E2	5.8e-177
ClaPPR188_PLS	1.2e-176
ClaPPR64_DYW	4.5e-176
ClaPPR395_E2	1.0e-174
ClaPPR113_E1	6.1e-173
ClaPPR54_E2	2.2e-172
ClaPPR22_E1	1.2e-171
ClaPPR245_E2	4.7e-171
ClaPPR145_P	2.9e-169
ClaPPR285_P	1.6e-168
ClaPPR312_E1	3.0e-168
ClaPPR84_P	9.2e-168
ClaPPR51_P	7.8e-166
ClaPPR186_P	8.5e-166
ClaPPR81_E2	1.6e-162
ClaPPR90_PLS	1.6e-161
ClaPPR37_E2	2.0e-161
ClaPPR198_P	9.7e-160
ClaPPR17_P	7.8e-159
ClaPPR218_P	1.6e-157
ClaPPR11_P	2.6e-157
ClaPPR401_PLS	3.3e-157
ClaPPR416_P	3.3e-157
ClaPPR63_P	6.7e-157
ClaPPR288_PLS	2.3e-156

Block Diagram

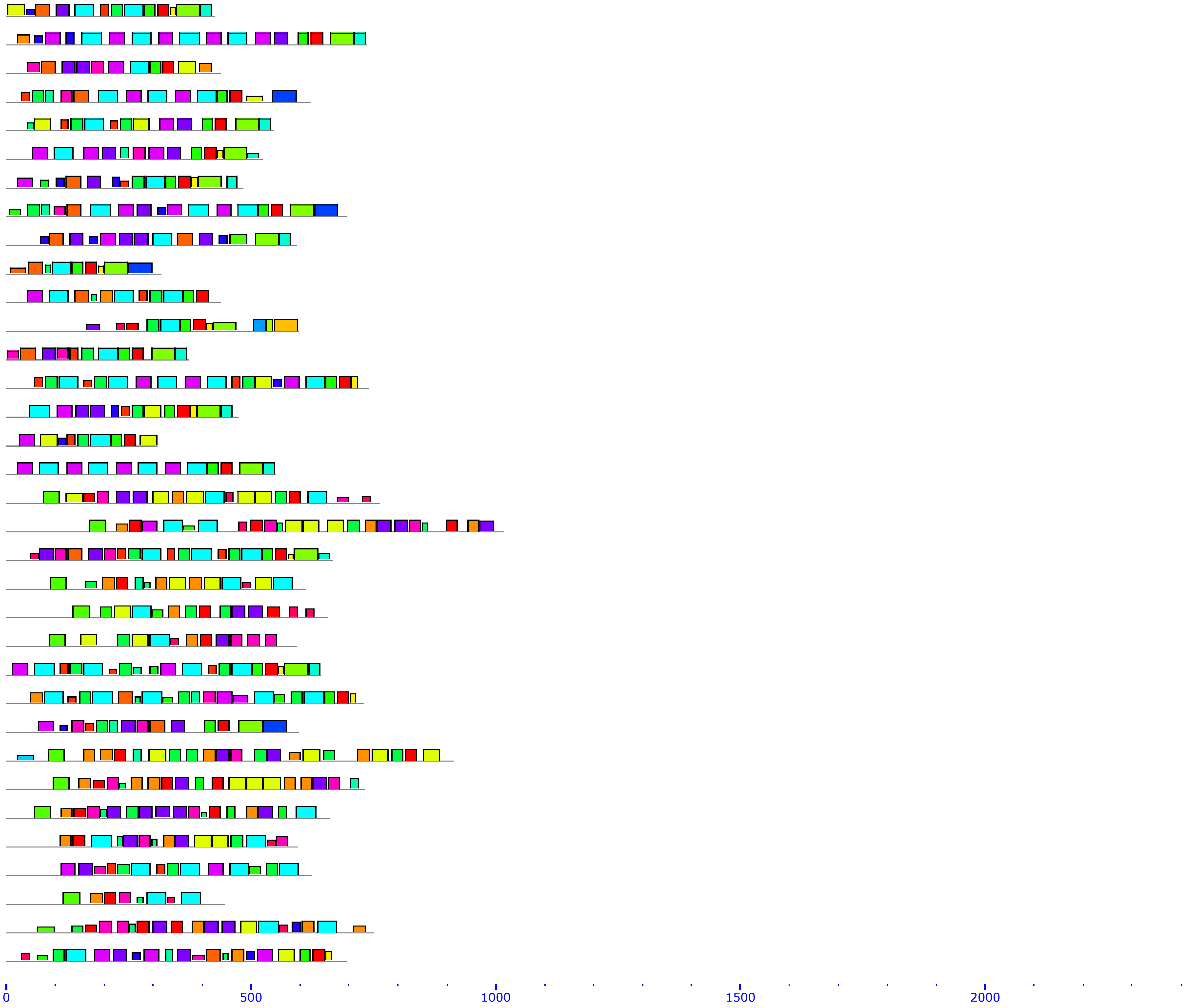

Sequence	E-value
ClaPPR380_P	1.4e-155
ClaPPR329_P	5.2e-155
ClaPPR299_PLS	3.8e-154
ClaPPR135_P	8.2e-154
ClaPPR344_P	1.3e-153
ClaPPR176_P	9.3e-153
ClaPPR80_P	9.6e-153
ClaPPR174_P	1.1e-152
ClaPPR391_P	1.4e-152
ClaPPR392_P	5.0e-152
ClaPPR61_P	7.7e-152
ClaPPR411_PLS	2.2e-151
ClaPPR350_PLS	1.0e-150
ClaPPR69_P	1.0e-150
ClaPPR294_P	4.8e-150
ClaPPR301_P	8.0e-150
ClaPPR78_P	2.7e-149
ClaPPR263_P	3.3e-148
ClaPPR154_P	2.6e-147
ClaPPR104_P	1.6e-146
ClaPPR173_P	2.1e-146
ClaPPR235_P	2.8e-146
ClaPPR326_P	7.3e-146
ClaPPR155_P	9.1e-146
ClaPPR295_P	3.5e-145
ClaPPR287_P	3.6e-145
ClaPPR195_P	2.1e-144
ClaPPR233_P	2.6e-144
ClaPPR193_P	3.2e-144
ClaPPR119_P	6.4e-144
ClaPPR172_P	6.7e-144
ClaPPR157_P	7.0e-144
ClaPPR9_PLS	1.1e-143
ClaPPR319_P	2.3e-143

Block Diagram

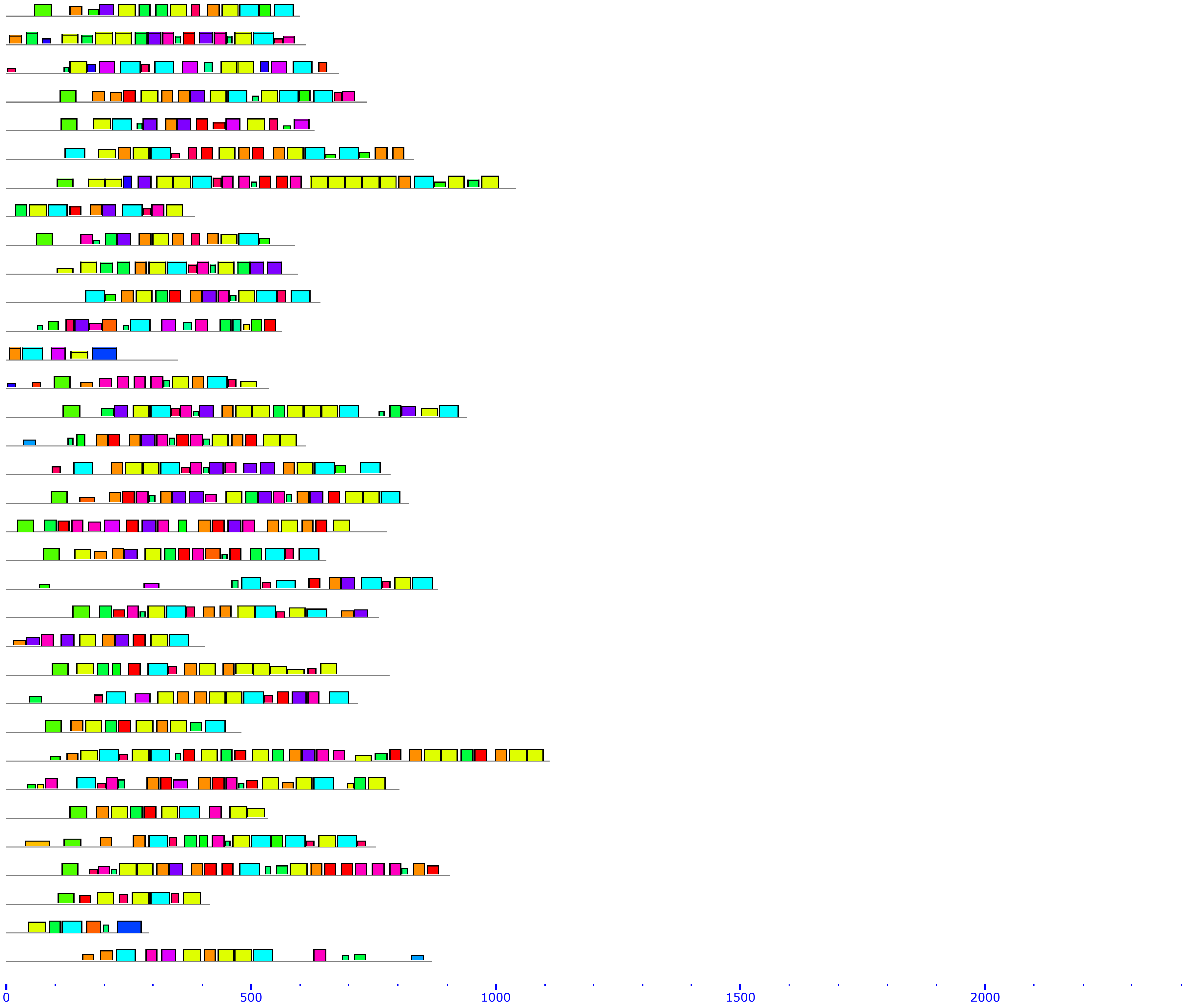

Sequence	E-value
ClaPPR19_P	3.4e-143
ClaPPR421_P	7.6e-143
ClaPPR345_P	1.5e-142
ClaPPR280_PLS	2.6e-142
ClaPPR348_P	3.7e-142
ClaPPR414_P	8.6e-142
ClaPPR368_P	4.2e-141
ClaPPR122_P	6.1e-141
ClaPPR236_P	6.8e-141
ClaPPR266_P	1.0e-140
ClaPPR48_P	4.7e-140
ClaPPR211_P	6.7e-140
ClaPPR117_P	4.5e-139
ClaPPR111_P	6.4e-137
ClaPPR144_P	1.1e-136
ClaPPR203_P	2.7e-136
ClaPPR46_P	1.9e-135
ClaPPR15_P	6.4e-135
ClaPPR33_P	7.7e-134
ClaPPR268_P	9.0e-134
ClaPPR65_P	1.6e-133
ClaPPR213_P	9.9e-133
ClaPPR258_P	1.2e-132
ClaPPR190_P	3.6e-131
ClaPPR6_P	4.3e-131
ClaPPR339_P	9.9e-131
ClaPPR234_P	2.2e-130
ClaPPR165_P	2.6e-129
ClaPPR76_P	4.4e-129
ClaPPR180_P	1.4e-128
ClaPPR408_P	2.2e-128
ClaPPR20_P	1.1e-127
ClaPPR38_P	2.1e-127
ClaPPR257_P	4.0e-127

Block Diagram

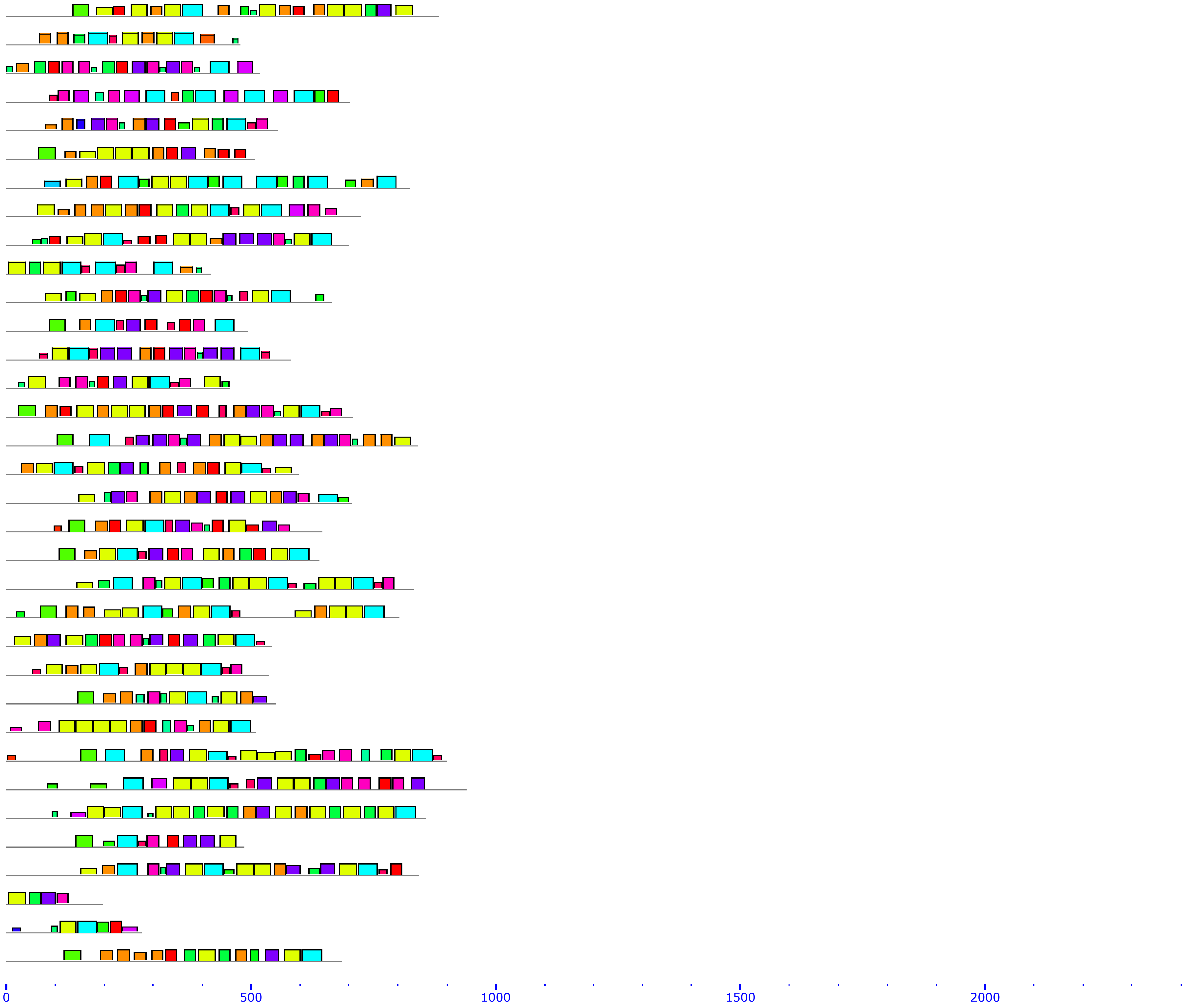

Sequence	E-value
ClaPPR223_P	1.2e-126
ClaPPR325_P	2.0e-126
ClaPPR27_P	3.7e-126
ClaPPR16_P	4.6e-126
ClaPPR118_P	5.1e-126
ClaPPR303_P	1.9e-125
ClaPPR406_P	1.3e-124
ClaPPR91_P	3.9e-124
ClaPPR361_P	2.0e-123
ClaPPR209_P	4.3e-123
ClaPPR417_P	8.6e-123
ClaPPR169_P	1.2e-122
ClaPPR137_P	6.7e-122
ClaPPR67_P	7.3e-122
ClaPPR346_P	1.1e-121
ClaPPR289_PLS	2.8e-121
ClaPPR68_P	1.2e-120
ClaPPR58_P	2.7e-120
ClaPPR418_P	3.0e-119
ClaPPR228_P	2.3e-118
ClaPPR261_P	3.8e-118
ClaPPR196_P	1.9e-117
ClaPPR222_P	6.6e-117
ClaPPR244_P	1.4e-116
ClaPPR129_P	1.7e-116
ClaPPR382_P	2.6e-116
ClaPPR402_P	3.6e-116
ClaPPR387_P	4.4e-115
ClaPPR254_P	4.6e-115
ClaPPR265_P	4.7e-115
ClaPPR277_P	1.1e-114
ClaPPR420_P	6.1e-114
ClaPPR18_P	2.8e-113
ClaPPR179_PLS	2.9e-113

Block Diagram

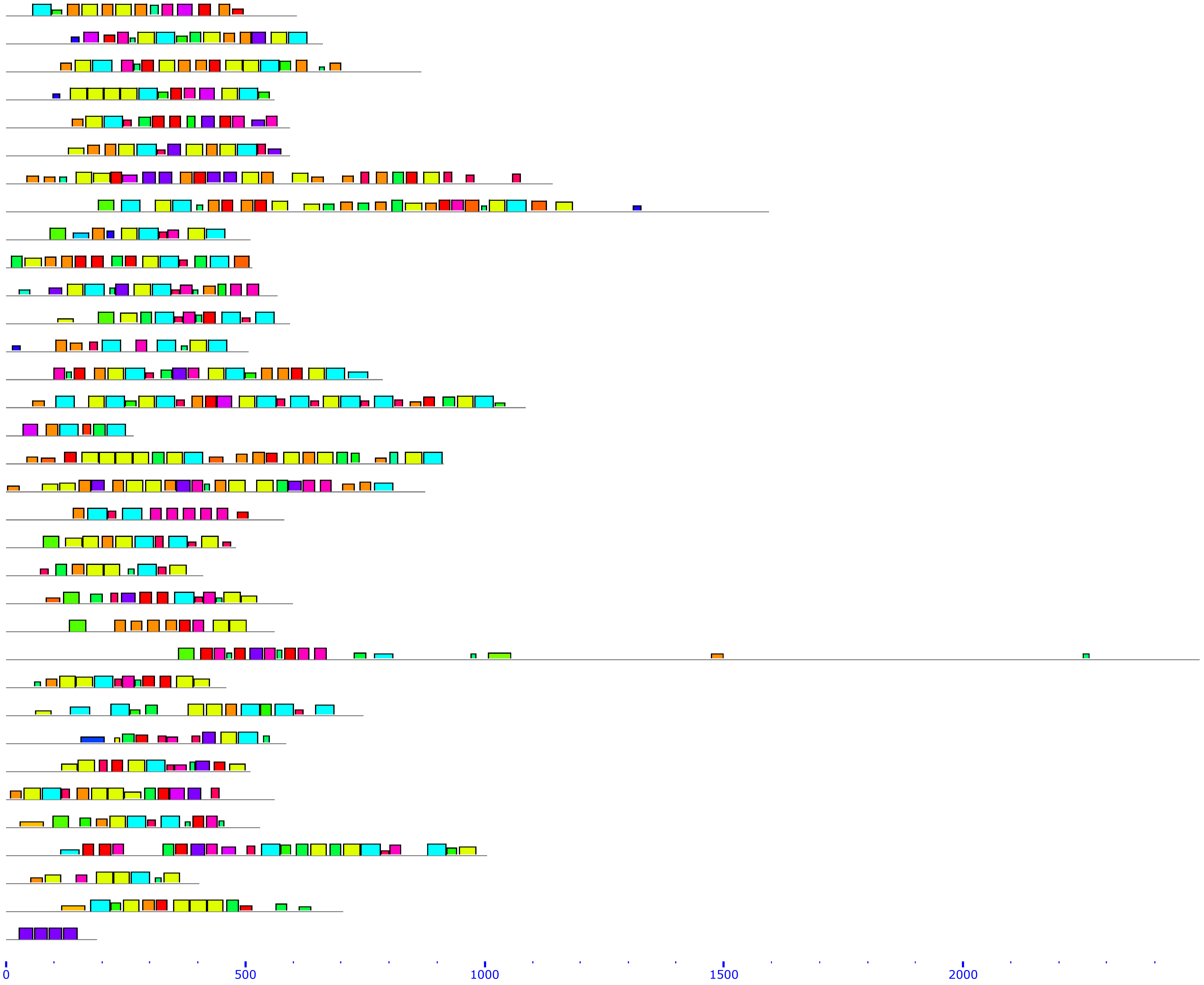

Sequence	E-value
ClaPPR279_PLS	3.1e-113
ClaPPR318_P	9.0e-113
ClaPPR200_P	2.6e-112
ClaPPR394_P	6.1e-112
ClaPPR36_P	1.0e-111
ClaPPR105_P	1.1e-111
ClaPPR328_P	2.1e-110
ClaPPR273_P	3.3e-110
ClaPPR385_P	1.5e-109
ClaPPR341_P	5.1e-109
ClaPPR7_P	7.0e-109
ClaPPR57_DYW	8.5e-109
ClaPPR5_P	9.9e-109
ClaPPR41_P	1.0e-108
ClaPPR376_P	1.1e-108
ClaPPR158_PLS	1.6e-108
ClaPPR413_P	8.1e-108
ClaPPR112_P	9.5e-108
ClaPPR148_P	3.4e-107
ClaPPR102_P	6.6e-107
ClaPPR352_P	9.1e-107
ClaPPR210_P	3.1e-106
ClaPPR66_P	4.2e-105
ClaPPR333_P	3.3e-103
ClaPPR334_P	3.6e-103
ClaPPR275_P	6.2e-103
ClaPPR10_P	3.4e-102
ClaPPR197_P	5.1e-102
ClaPPR156_P	4.0e-101
ClaPPR183_P	1.5e-100
ClaPPR99_P	5.1e-100
ClaPPR83_P	1.3e-99
ClaPPR327_P	6.0e-99
ClaPPR322_P	3.5e-98

Block Diagram

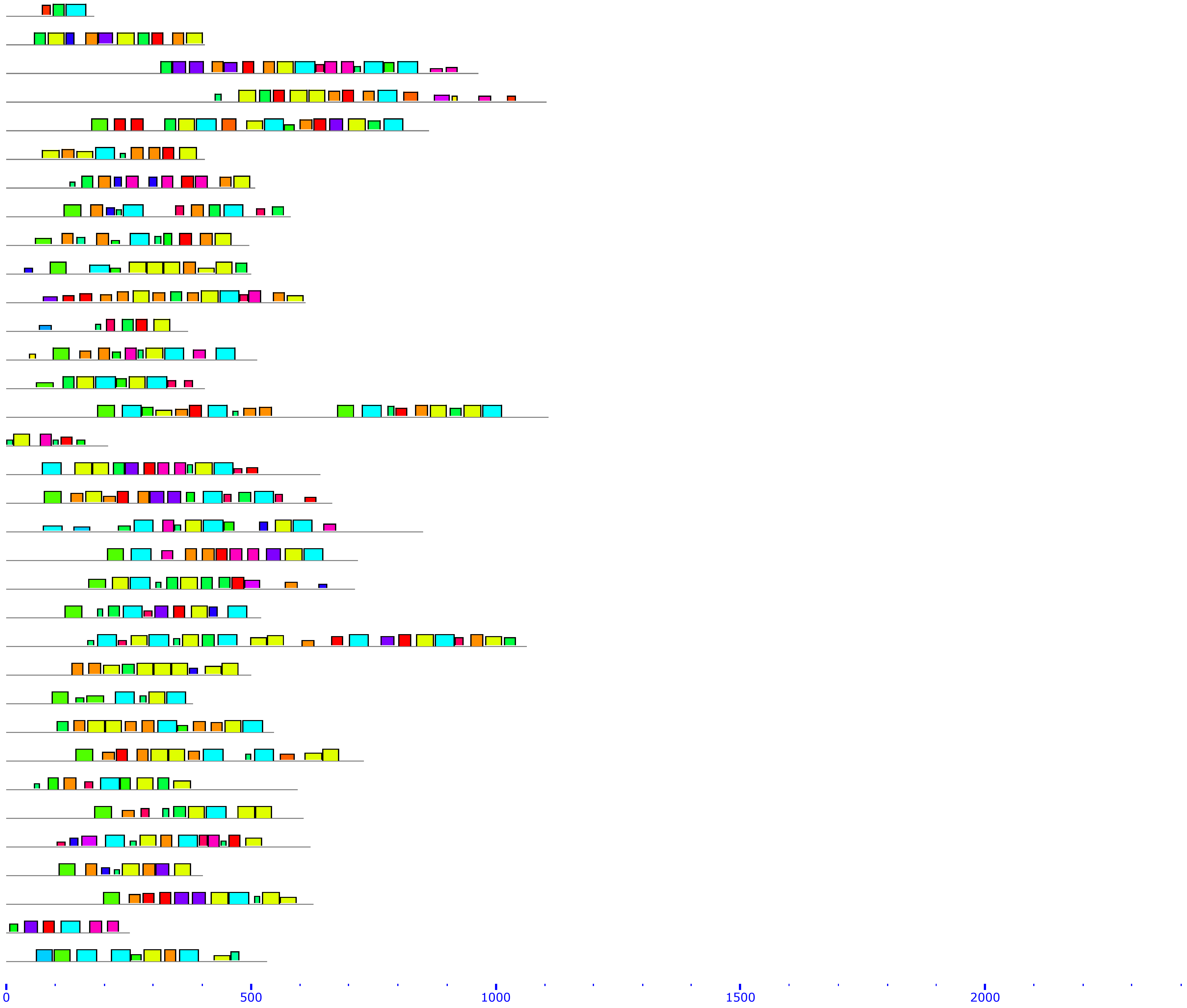

Sequence	E-value
ClaPPR283_P	3.9e-97
ClaPPR422_P	2.4e-96
ClaPPR255_P	4.7e-96
ClaPPR367_P	5.6e-96
ClaPPR267_P	1.1e-95
ClaPPR247_P	3.0e-94
ClaPPR217_P	8.5e-94
ClaPPR304_P	1.2e-93
ClaPPR13_P	2.1e-93
ClaPPR34_P	4.7e-93
ClaPPR332_P	1.3e-92
ClaPPR396_P	2.2e-90
ClaPPR75_P	2.4e-90
ClaPPR317_P	3.9e-90
ClaPPR14_P	1.9e-88
ClaPPR225_P	2.6e-88
ClaPPR204_P	3.7e-88
ClaPPR239_P	5.2e-88
ClaPPR356_P	1.6e-86
ClaPPR357_P	2.4e-86
ClaPPR82_P	2.5e-86
ClaPPR400_P	1.6e-85
ClaPPR163_P	2.2e-85
ClaPPR363_P	3.1e-85
ClaPPR114_P	3.5e-85
ClaPPR370_P	9.8e-85
ClaPPR215_PLS	1.3e-84
ClaPPR358_P	1.1e-83
ClaPPR212_P	8.0e-82
ClaPPR371_P	8.8e-82
ClaPPR107_P	1.0e-81
ClaPPR311_P	1.0e-81
ClaPPR256_P	8.5e-80
ClaPPR364_P	7.9e-79

Block Diagram

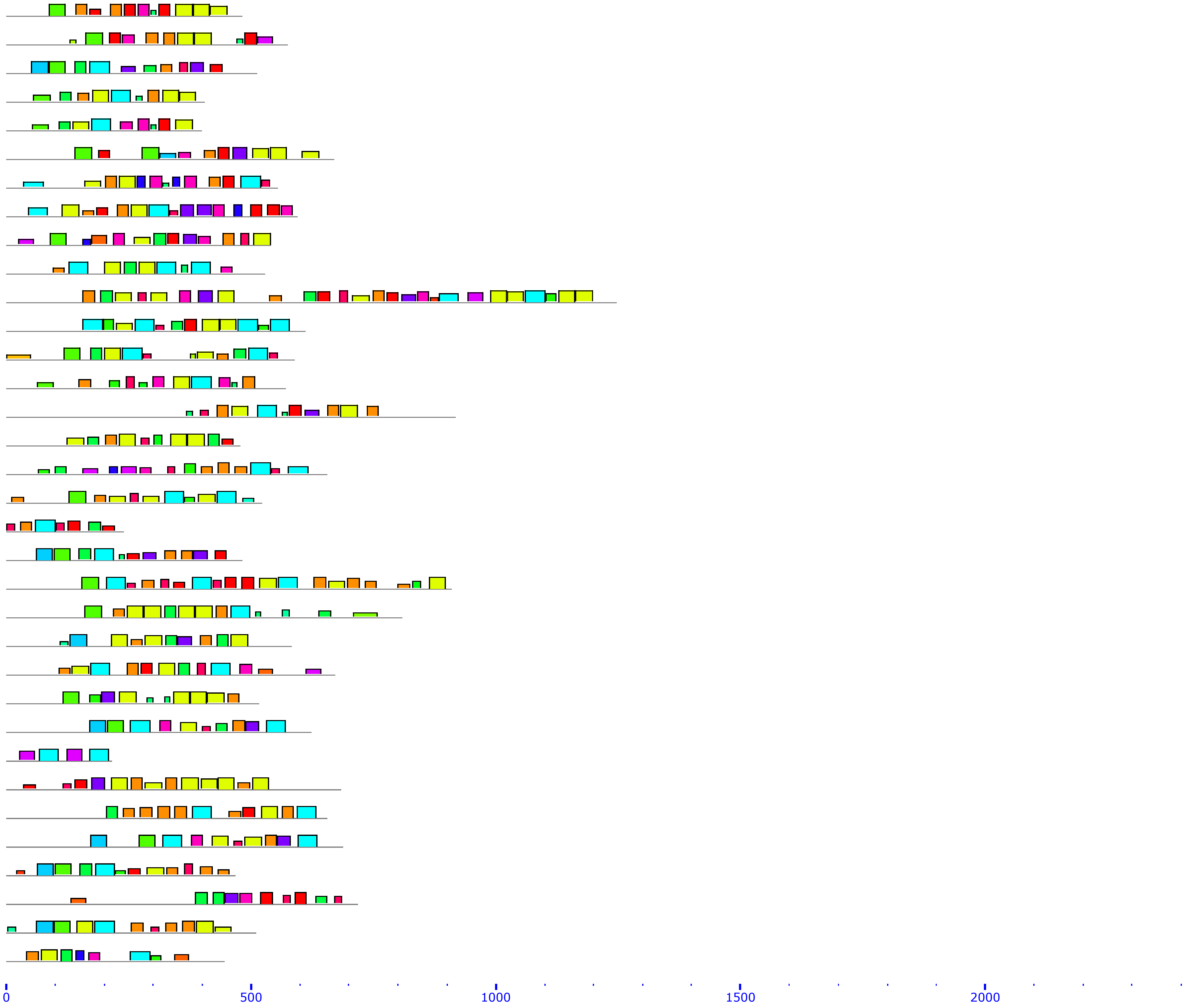

Sequence	E-value
ClaPPR106_P	3.0e-78
ClaPPR241_P	7.2e-78
ClaPPR412_P	1.1e-76
ClaPPR93_P	2.2e-75
ClaPPR297_P	3.9e-75
ClaPPR377_P	2.6e-74
ClaPPR160_P	1.4e-72
ClaPPR397_P	1.9e-71
ClaPPR192_P	1.1e-70
ClaPPR338_P	8.9e-69
ClaPPR208_P	6.1e-68
ClaPPR103_P	9.9e-66
ClaPPR276_P	1.1e-65
ClaPPR32_P	3.0e-63
ClaPPR298_P	3.6e-62
ClaPPR300_P	1.3e-60
ClaPPR331_P	2.7e-58
ClaPPR355_P	2.8e-57
ClaPPR324_P	3.9e-56
ClaPPR405_P	3.1e-55
ClaPPR252_P	6.3e-55
ClaPPR26_P	2.7e-51
ClaPPR336_P	3.5e-45
ClaPPR393_E2	3.5e-45
ClaPPR72_P	7.7e-45
ClaPPR53_DYW	2.9e-37
ClaPPR224_E2	3.0e-32
ClaPPR121_PLS	1.1e-26

Block Diagram

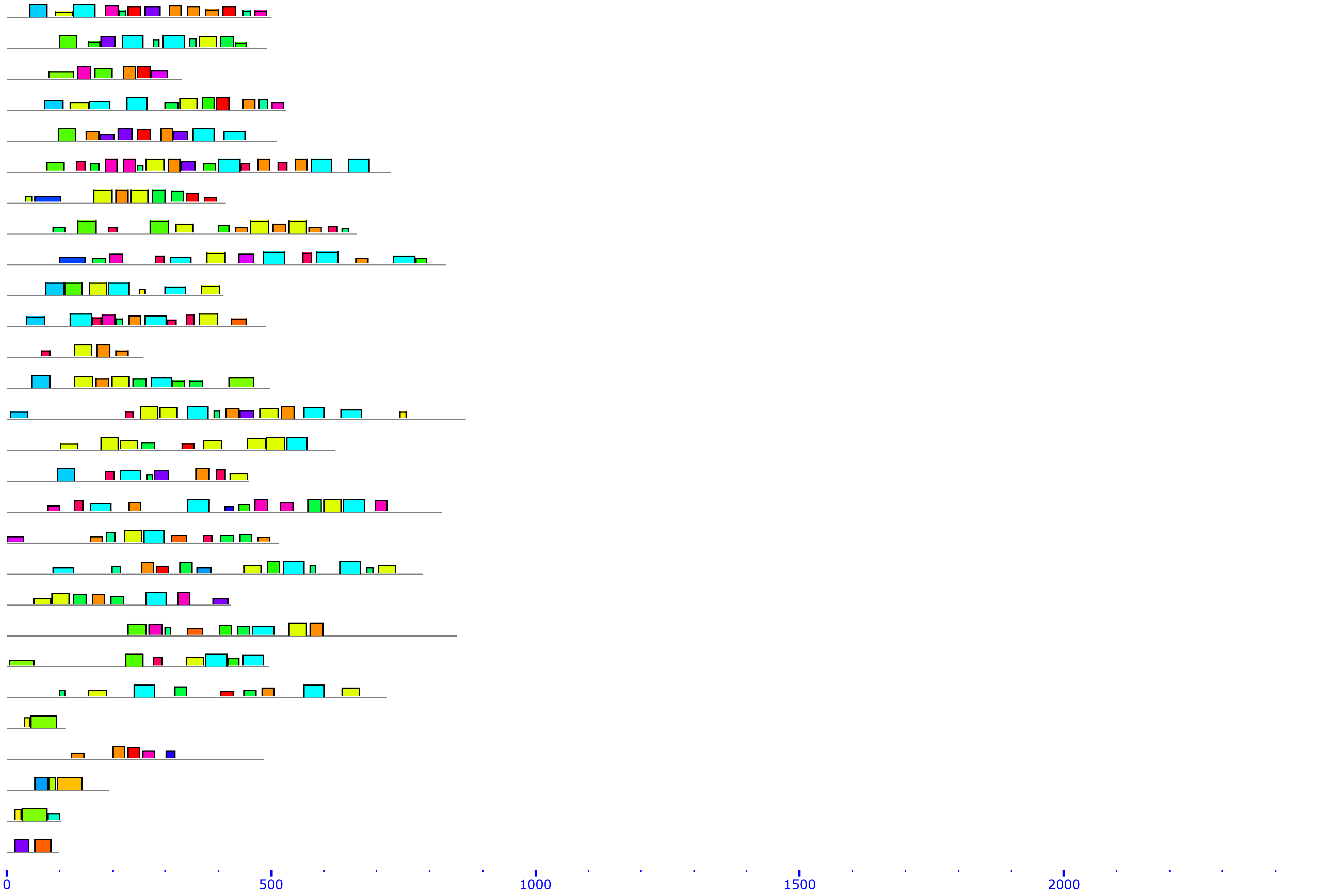

GIEPBVEHYGCLVDLLGRAGRLEEA
PBVVSWNAMISGYAKHGRGEEALELFEEMKESGVKPDEVTF
VWGALLGACRIHGNVELGERAAEKLLELEPENSGNYVLLSNIYASAGRW
FDEMPEPBVVSWNALISGYAKNGRVEEAL
IRIVKNLRVCGDCHSAIKLISKIVGREIIVRDRNRFHHFKBGSCSCGDYW
VYNALIDMYAKCGSLEEARKVFDEM
EDVAKVRKLMKERGVKKEPGCSWIEVNGKVHEFVAGDKSHPQSEEIYEKL
FEEMLEEGVKPBEVTFNSLJSACAK
ALELGKQIHGYMIKLGLEPBVFVYNALIDMY
PEPBVVTWNSLISGYCKNGLLEEAJELFEEMLEKG
VGVLSACSHAGLVEEGRKYFNSM
PBVVSWNALISGYAKNGD
YVPDTSFVLHDVEEEEKEEALSYHSEK
KPBEFTYNSVLSACAKLG
KQIHAQMIKSGLEPBVFVYNSLIDMYAKCGSJ
ALIDGYCKNGKLEEALKL
ALELGKQIHGYIIKSGLE
SYNILISGYCKNGRVDEALKLFDEM
ELIESMPIEPBAV
LAIAFGLJSTPPG
ALEFFEWAGKQPGFKHTPETYNIMJDILGRARGFK
EPBVVTYNSLISGLCKNG
GDVDEARKLFDEM
DDVEKVRELMRERGVKKEPGYSWI
SVVIVLBQWVEEGKEIKKYELSRIVKELRKYRRYG
