# Supplementary figures and images for: A Genome-Wide Analysis of the Pentatricopeptide Repeat (PPR) Gene Family and PPR-Derived Markers for Flesh Color in Watermelon (Citrullus lanatus)
Source: Genes (Basel). 2020 Sep 24;11(10):1125. doi: 10.3390/genes11101125 (PMC7650700; doi:10.3390/genes11101125)

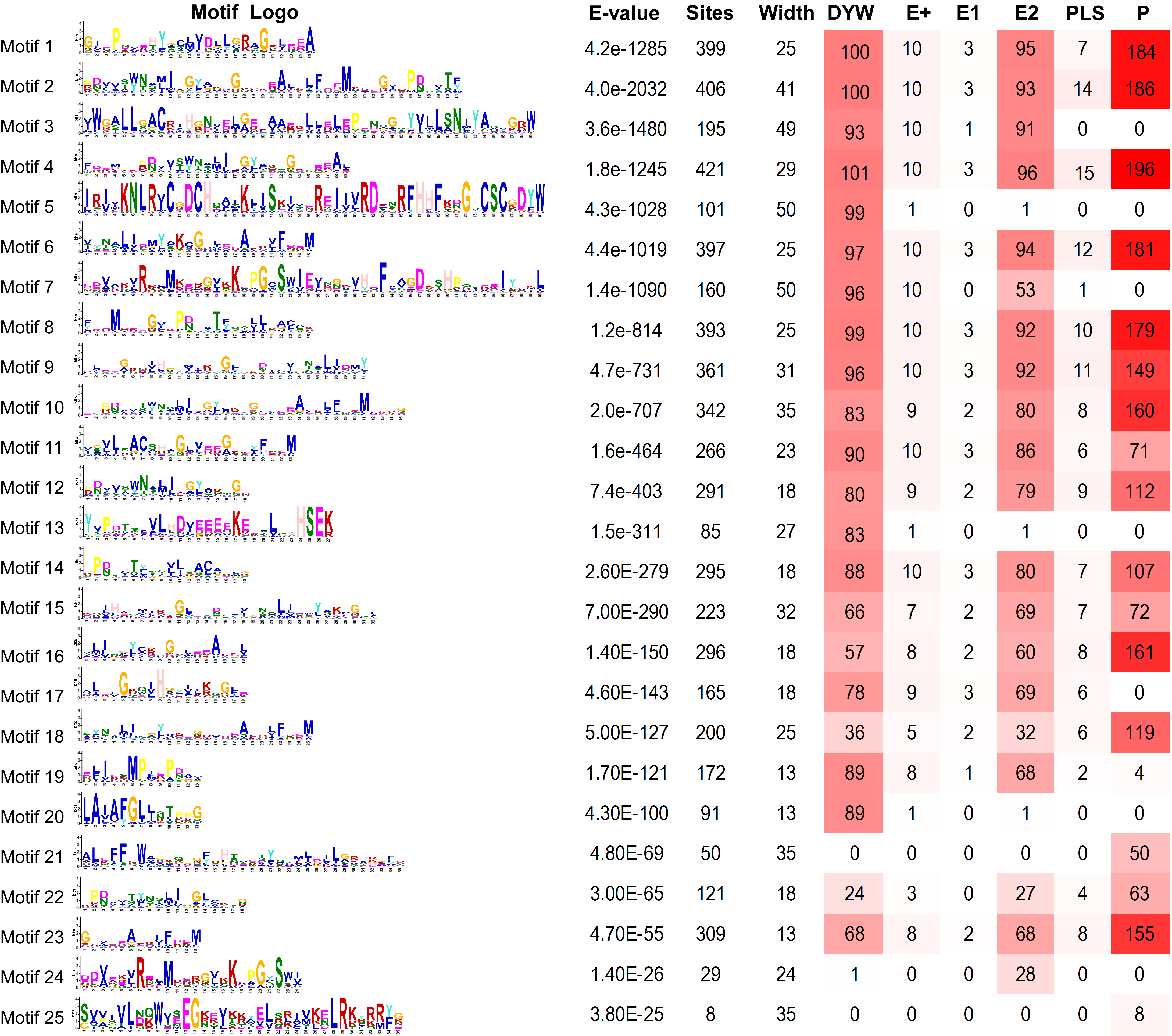

Supplement: Supplementary file 1 [file genes-11-01125-s001.zip › genes_925536-Supplementary/Supplemtary Figures/Figure S2 .tif]

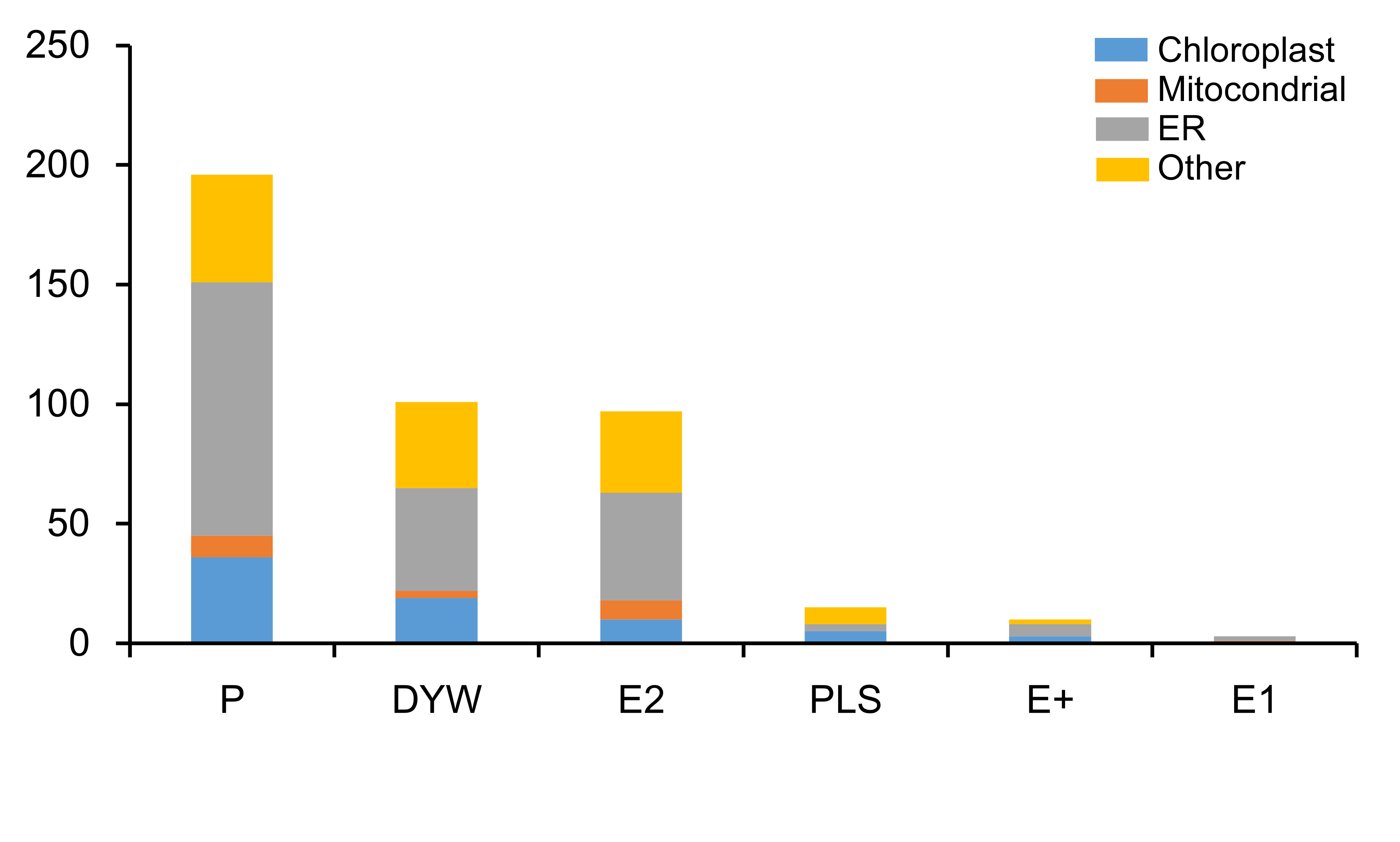

Supplement: Supplementary file 1 [file genes-11-01125-s001.zip › genes_925536-Supplementary/Supplemtary Figures/Figure S3 .tif]

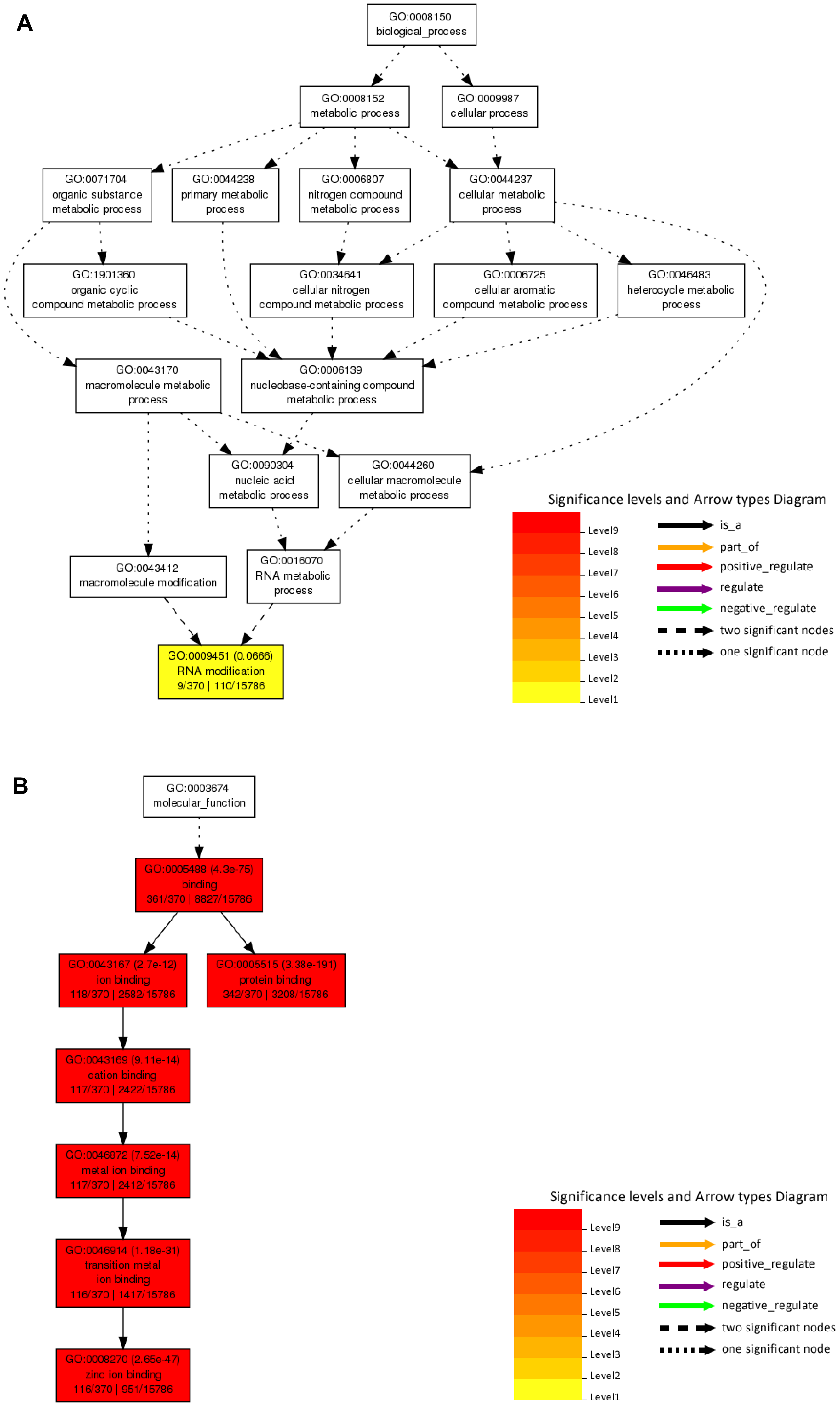

Supplement: Supplementary file 1 [file genes-11-01125-s001.zip › genes_925536-Supplementary/Supplemtary Figures/Figure S4.tif]

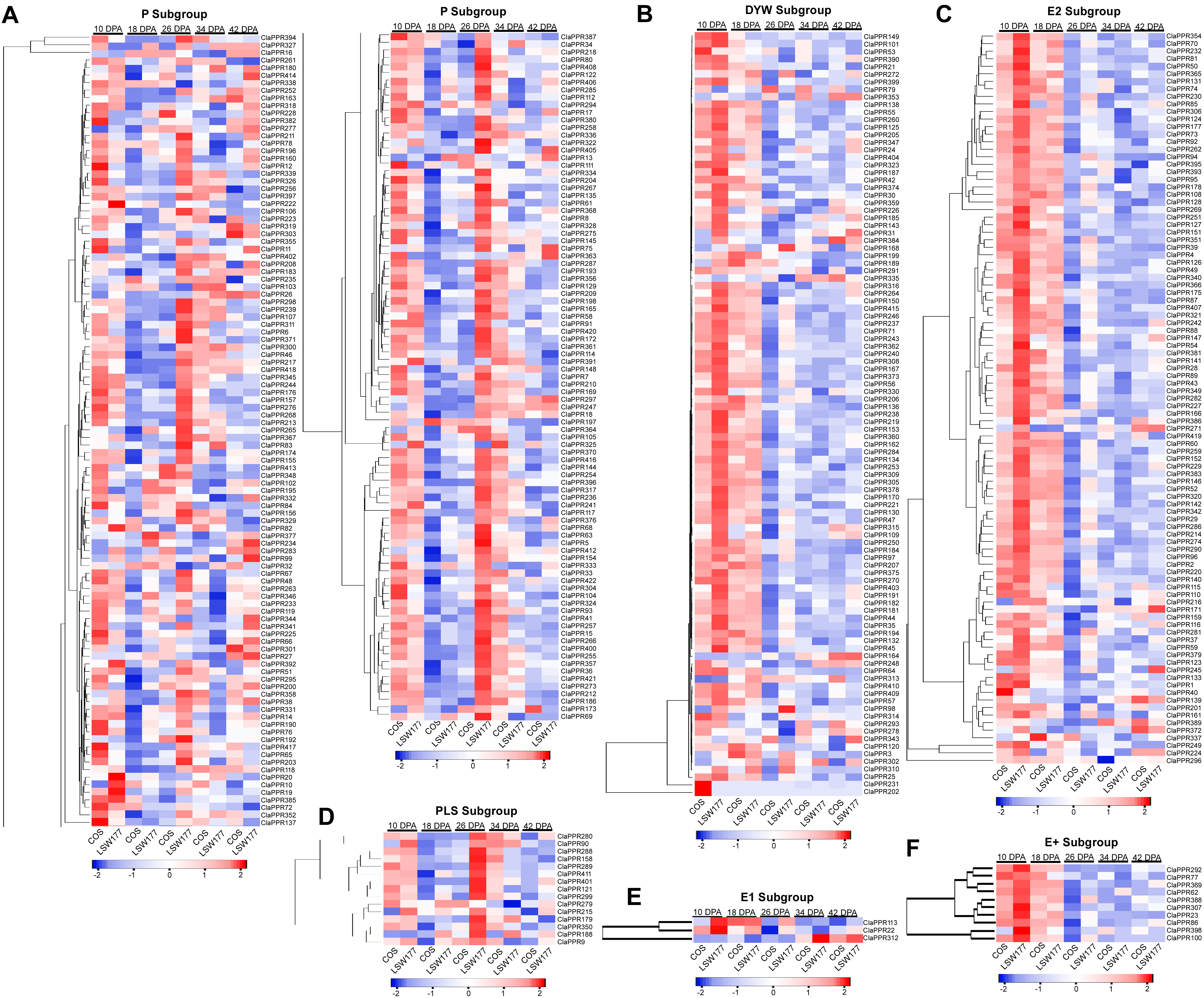

Supplement: Supplementary file 1 [file genes-11-01125-s001.zip › genes_925536-Supplementary/Supplemtary Figures/Figure S5.tif]
